# Streptolysin S is required for *Streptococcus pyogenes* nasopharyngeal and skin infection in HLA-transgenic mice

**DOI:** 10.1371/journal.ppat.1012072

**Published:** 2024-03-07

**Authors:** Blake A. Shannon, Jacklyn R. Hurst, Ronald S. Flannagan, Heather C. Craig, Aanchal Rishi, Katherine J. Kasper, Stephen W. Tuffs, David E. Heinrichs, John K. McCormick

**Affiliations:** Department of Microbiology and Immunology, University of Western Ontario, London, Ontario, Canada; Lunds universitet Medicinska fakulteten, SWEDEN

## Abstract

*Streptococcus pyogenes* is a human-specific pathogen that commonly colonizes the upper respiratory tract and skin, causing a wide variety of diseases ranging from pharyngitis to necrotizing fasciitis and toxic shock syndrome. *S*. *pyogenes* has a repertoire of secreted virulence factors that promote infection and evasion of the host immune system including the cytolysins streptolysin O (SLO) and streptolysin S (SLS). *S*. *pyogenes* does not naturally infect the upper respiratory tract of mice although mice transgenic for MHC class II human leukocyte antigens (HLA) become highly susceptible. Here we used HLA-transgenic mice to assess the role of both SLO and SLS during both nasopharyngeal and skin infection. Using *S*. *pyogenes* MGAS8232 as a model strain, we found that an SLS-deficient strain exhibited a 100-fold reduction in bacterial recovery from the nasopharynx and a 10-fold reduction in bacterial burden in the skin, whereas an SLO-deficient strain did not exhibit any infection defects in these models. Furthermore, depletion of neutrophils significantly restored the bacterial burden of the SLS-deficient bacteria in skin, but not in the nasopharynx. In mice nasally infected with the wildtype *S*. *pyogenes*, there was a marked change in localization of the tight junction protein ZO-1 at the site of infection, demonstrating damage to the nasal epithelia that was absent in mice infected with the SLS-deficient strain. Overall, we conclude that SLS is required for the establishment of nasopharyngeal infection and skin infection in HLA-transgenic mice by *S*. *pyogenes* MGAS8232 and provide evidence that SLS contributes to nasopharyngeal infection through the localized destruction of nasal epithelia.

## Author summary

*Streptococcus pyogenes* is a human-specific bacteria that causes a massive global burden of disease, ranging from mild infections such as strep throat, to more serious diseases such as flesh-eating disease and toxic shock syndrome. *S*. *pyogenes* has two toxins, streptolysin O (SLO) and streptolysin S (SLS), which contribute to disease progression by causing cell death. The streptolysins have been extensively studied in mouse models of skin and invasive infections; however, their role in nasal infection is understudied. We found that removal of the SLS toxin from the bacteria caused a significant reduction in the infection burden of nasal and skin tissues in mice. Interestingly, the SLO toxin was not a major contributor to disease in either of these infection models. We provide evidence suggesting that SLS targets the mucosal barrier in the nasal tissues of mice to help cause nasal infection. We also show that SLS helps cause skin infection by protecting the bacteria from neutrophils. Overall, strep throat and skin infections are the main *S*. *pyogenes* diseases, and this work has the potential to guide the development of therapies to prevent and treat *S*. *pyogenes* infections by targeting SLS.

## Introduction

*Streptococcus pyogenes* (also commonly referred to as the Group A *Streptococcus*) is a Gram-positive bacterial pathogen placed among the top ten causes of infection-related mortality worldwide [1]. This pathogen has strict host tropism, with humans being the only natural reservoir [2]. *S*. *pyogenes* primarily subsists as an asymptomatic colonizer of the upper respiratory tract and skin, with one meta-analysis finding the prevalence of asymptomatic carriage to be between 5–12% across school-aged children in developed countries [3]. Nevertheless, colonization by the bacteria may result in acute infections such as pharyngitis and impetigo, or more serious manifestations such as necrotizing fasciitis and toxic shock syndrome. Additionally, repeated pharyngeal infections with *S*. *pyogenes* can lead to acute rheumatic fever, which can progress to rheumatic heart disease [1]. Overall, the burden of disease by *S*. *pyogenes* translates to over 500,000 annual deaths worldwide, with the majority of estimated deaths due to complications of rheumatic heart disease [1].

*S*. *pyogenes* encodes a repertoire of virulence factors that promote pathogenesis, including two cytolytic toxins known as streptolysin O (SLO) and streptolysin S (SLS) [1]. The *slo* gene encodes SLO, an oxygen-labile, cholesterol-dependent cytolysin that oligomerizes on the surface of host cells upon binding to cholesterol, forming a large pore that ultimately results in host cell death [4–6]. SLO has a broad spectrum of activity and can target many cell types including erythrocytes, macrophages, neutrophils, and keratinocytes [7–10]. The other streptococcal cytolysin, SLS, is a small, oxygen-stable hemolysin responsible for the characteristic β-hemolysis of *S*. *pyogenes* seen on blood agar plates [11]. SLS also targets and disrupts the function of multiple cell types including erythrocytes, macrophages, neutrophils, and keratinocytes [11–14]. Specifically, SLS has recently been shown to interact with ion transporters on the cell surface of erythrocytes and keratinocytes to induce cytotoxicity [15,16]. Furthermore, SLS can cause the disruption of the epithelial barrier through the degradation of tight junctions *in vitro* [17].

Both SLO and SLS have been extensively studied in the context of sepsis and invasive skin and soft tissue disease, with many studies concluding that both cytolysins are crucial for invasive streptococcal disease [18–24]. However, pharyngeal infection represents the predominant manifestation of disease, and the majority of deaths attributed to streptococcal infections are a result of rheumatic heart disease, caused by repetitive bouts of streptococcal pharyngitis. While SLO has been shown to promote nasopharyngeal infection in an *emm12 S*. *pyogenes* strain [25], SLS has yet to be studied in the context of nasopharyngeal infections.

Herein, we use an established murine model of nasopharyngeal infection that uses transgenic mice on a C57Bl/6 background expressing HLA-DR4 and HLA-DQ8 (herein referred to as B6_HLA_ mice) [26]. We demonstrate that SLS is essential for high bacterial burdens in the nasopharynx and skin of B6_HLA_ mice, while SLO does not significantly contribute to *S*. *pyogenes* nasopharyngeal and skin infection. Neutrophil-depletion partially restores the low burden of the SLS-deficient strain during skin infection, yet during nasopharyngeal infection, the SLS-deficient strain maintains its infection defect in neutrophil-depleted mice. Cytokine and immunohistological analysis of infected nasal turbinates suggest that SLS promotes inflammation by disrupting the nasal epithelia to advance experimental nasopharyngeal infection.

## Results

### Genetic deletion of the *slo* or *sag* operon result in a loss of hemolytic activity

**Fig 1:**
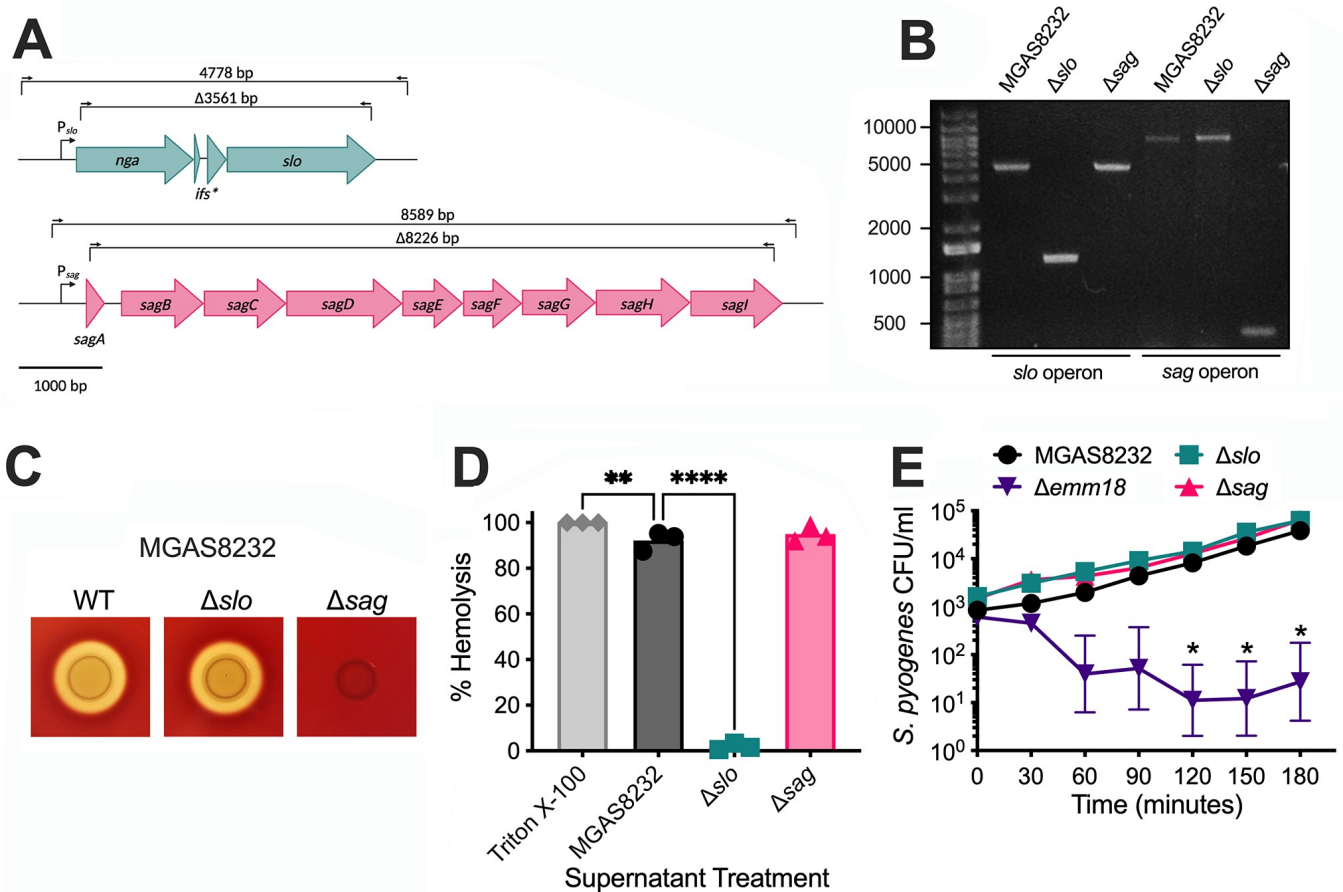
Deletion of the *slo* operon and the *sag* operon from the *S*. *pyogenes* MGAS8232 genome. **(A)** Genetic deletions of the *slo* operon (teal) or the *sag* operon (pink) were generated in *S*. *pyogenes* MGAS8232. The gene *nga* encodes an NADase, *slo* encodes the pore forming monomer and ifs* encodes a truncated form of the NADase inhibitor. The gene *sagA* encodes the unmodified SLS peptide, *sagB-D* encode post-translational modification machinery, *sagE* encodes an immunity-like protein and *sagG-I* encode transport machinery. The role of *sagF* is unknown. **(B)** PCR amplification for the *slo* or *sag* operon from *S*. *pyogenes* MGAS8232 wildtype, Δ*slo*, or Δ*sag* strains. **(C)** *S*. *pyogenes* MGAS8232 strains grown on TSA with 5% sheep’s blood. **(D)** Human red blood cell hemolysis by overnight *S*. *pyogenes* culture supernatants. **(E)** *S*. *pyogenes* survival in whole human blood. Blood was inoculated with 10^3^ CFU of bacteria and grown for 180 minutes, with bacterial concentration measured every 30 minutes. (* p < 0.01; ** p < 0.01; **** p < 0.0001).

To begin to examine the streptolysins during acute *S*. *pyogenes* infections, genetic deletions of the *slo* operon, including the *slo*, *ifs* and *nga* genes [27], or the *sag* operon encoding SLS biosynthesis and secretion [23], were generated in *S*. *pyogenes* strain MGAS8232 (**Fig 1A and 1B**), which was isolated from a patient with acute rheumatic fever [28]. Although *S*. *pyogenes* has two hemolysins, SLS is responsible for the characteristic β-hemolysis of *S*. *pyogenes* on blood agar plates. To confirm the loss of SLS-dependent hemolytic activity, strains were plated on tryptic soy agar (TSA) containing 5% sheep blood. A complete loss of hemolysis of the *S*. *pyogenes* Δ*sag* strain was observed, highlighting the genetic deletion of the *sag* operon (**Fig 1C**). To examine hemolytic activity by SLO, a red blood cell (RBC) hemolysis assay was performed using purified RBCs from human donors. When RBCs were treated with overnight culture supernatants, there was a significant decrease in hemolysis by the *S*. *pyogenes* Δ*slo* culture supernatant, but not the wildtype or Δ*sag* supernatant (**Fig 1D**). Of note, SLS-induced hemolysis was not observed in this assay since it requires the addition of a co-factor such as lipoteichoic acid for hemolytic activity in culture supernatants [11]. Therefore, the amount of SLO in wildtype and Δ*sag* culture supernatants was sufficient to induce the complete lysis of RBCs, and the lack of hemolysis by the Δ*slo* culture supernatant highlights the deletion of the SLO toxin. Furthermore, we found no growth differences among the three strains when grown in THY medium (**S1A Fig**). To assess the fitness of these strains, whole human blood was inoculated with ~1000 CFU ml^-1^ of each strain. There was no significant difference in bacterial growth of the streptolysin-deficient strains in whole human blood, whereas an *emm18* deletion strain grew poorly, demonstrating that both SLO and SLS are not required for survival in whole human blood in *S*. *pyogenes* MGAS8232 (**Fig 1E**). Together, these data confirm the genetic and functional deletion of the SLO and the SLS toxins from *S*. *pyogenes* MGAS8232.

### SLS is crucial for the establishment of *S*. *pyogenes* nasopharyngeal infection

**Fig 2:**
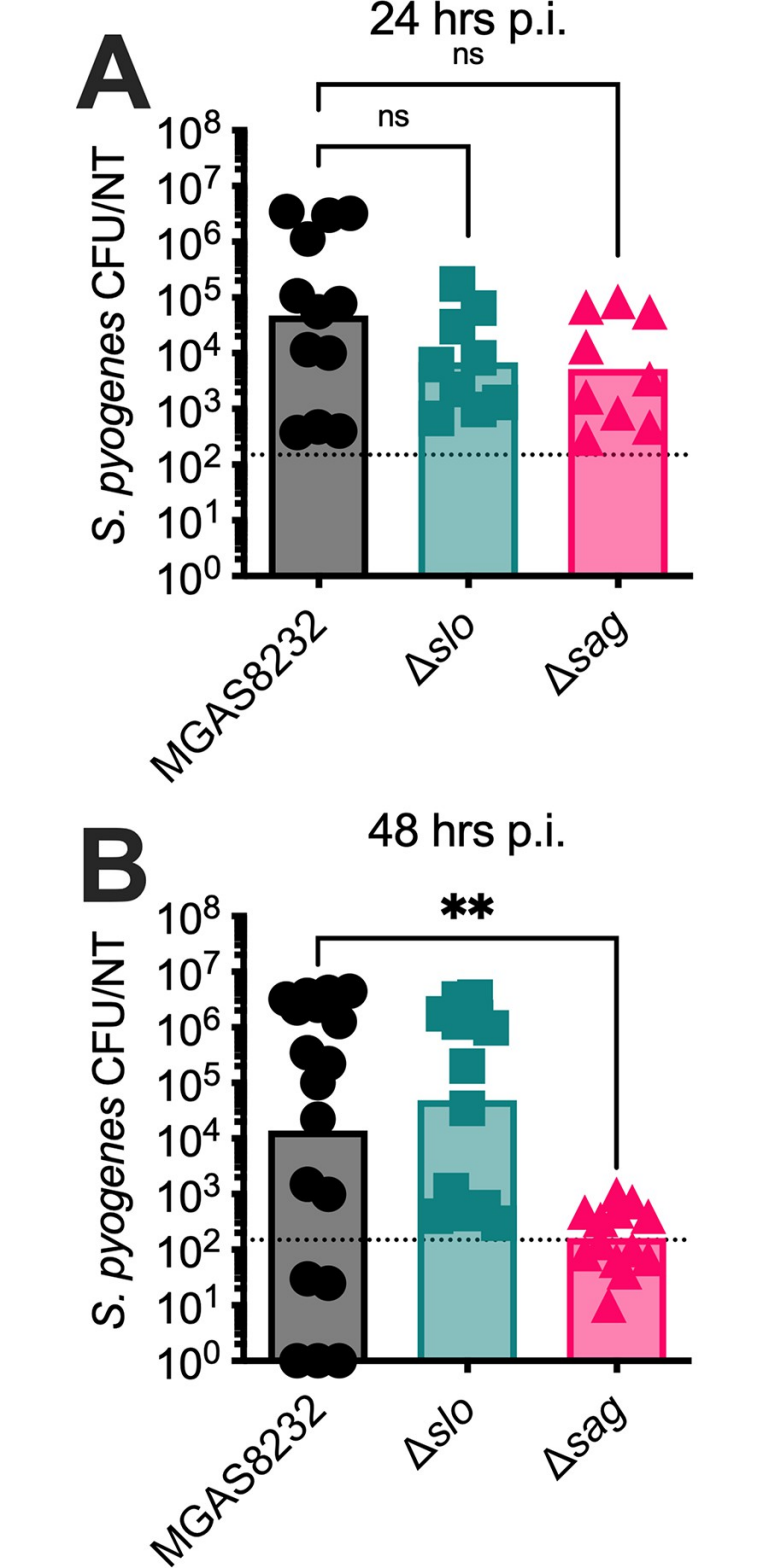
SLS-deficient *S*. *pyogenes* MGAS8232 exhibits impaired infection of the nasopharynx 48 hours post-infection. 8–12-week-old B6_HLA_ mice were nasally inoculated with ~1×10^8^ CFU of either the wildtype *S*. *pyogenes* MGAS8232, Δ*slo*, or Δ*sag* strain. Bacterial burden in the nasal turbinates was determined **(A)** 24 hours or **(B)** 48 hours post-inoculation. Data points represent individual mice and bars represent the geometric mean. Dotted line represents the limit of detection (10^2.17^). Statistical analyses comparing mutant infections to the wildtype MGAS8232 infection was done using a Kruskal-Wallis one-way ANOVA test and an uncorrected Dunn’s test for multiple comparisons (** p < 0.01).

While *S*. *pyogenes* can cause a wide array of diseases in humans, it most commonly causes acute nasopharyngeal infection, also known as strep throat [1]. The human-specific tropism of *S*. *pyogenes* warrants the use of animal models that are susceptible to infection by the bacteria. We previously developed a murine model of nasopharyngeal infection using transgenic mice expressing human MHC class II molecules (B6_HLA_ mice). These mice are more receptive to infection due to an increased susceptibility to bacterial superantigens [26], and we used this model to investigate the role of the streptolysins in nasopharyngeal infection. B6_HLA_ mice were infected with wildtype or streptolysin-deficient *S*. *pyogenes* strains, and bacterial recovery from the nasopharynx was determined 24 and 48 hours post-infection. Although there were no significant differences among infected mice 24 hours post-infection, there was a remarkable 100-fold decrease in bacterial burden in mice infected with the *S*. *pyogenes* Δ*sag* strain 48 hours post-infection (**Fig 2A and 2B**). Interestingly, this reduction in infection burden was not observed in mice infected with the *S*. *pyogenes* Δs*lo* strain at either timepoint (**Fig 2B**). As the SpeA superantigen is also known to be essential for productive nasopharyngeal infection [26], we confirmed that SpeA was expressed normally from each of the three strains (**S1B Fig**) These data suggest that SLS is essential for the establishment of nasopharyngeal infection, while SLO is not required for infection.

### SLS significantly contributes to skin infection pathology and bacterial burden in B6_HLA_ mice

**Fig 3:**
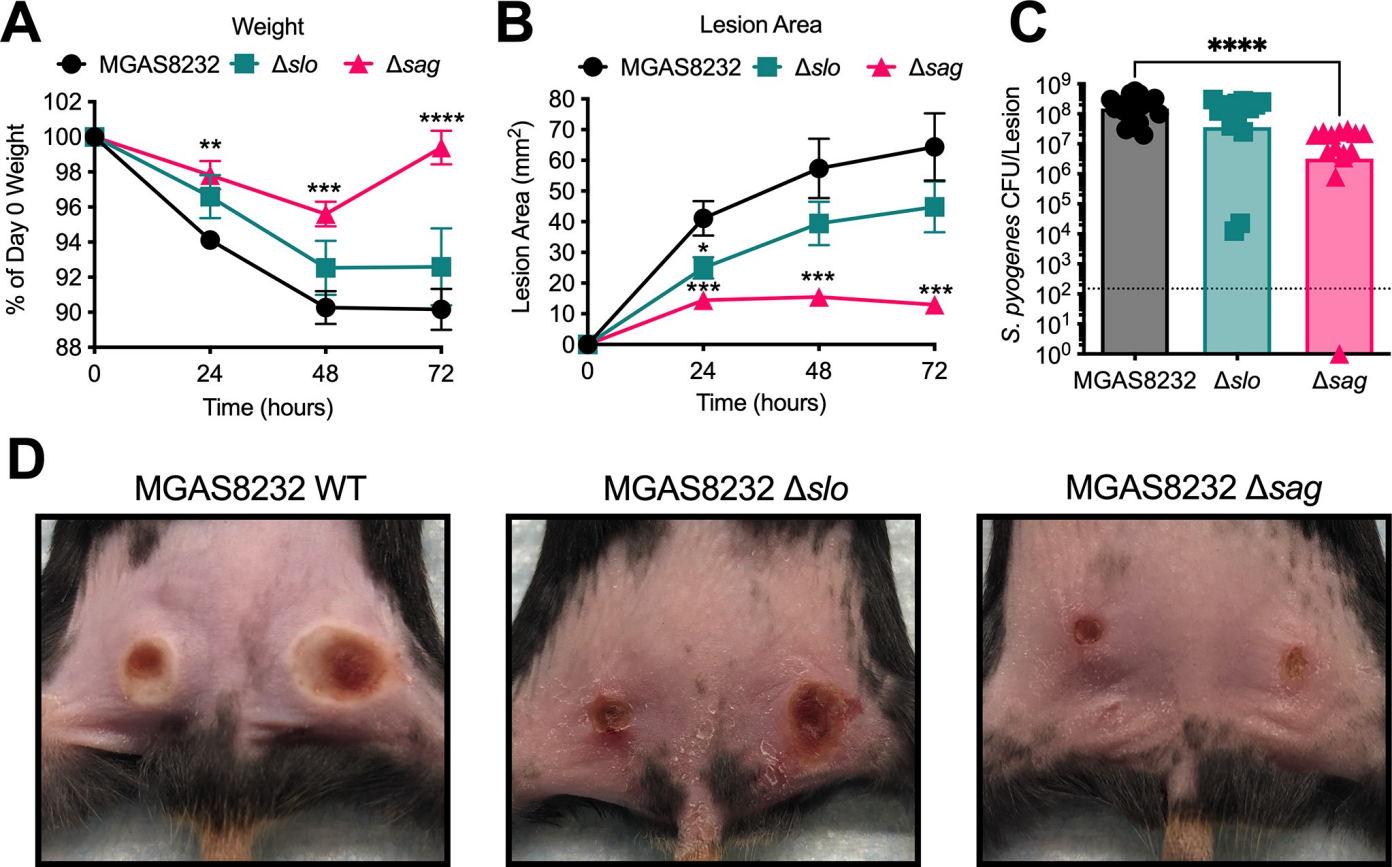
SLS promotes skin infection in HLA-transgenic mice. 8–12-week-old B6_HLA_ mice were infected with 5 × 10^7^ CFU of *S*. *pyogenes*, with 2.5 × 10^7^ bacteria injected into each flank. **(A)** Weights are presented as a percent of weight on day 0. **(B)** Lesions were measured using calipers at 24, 48, and 72 hours post-infection, and lesion area was calculated using two measurements perpendicular to each other. Data points represent mean +/- SE. Significance was determined by comparing mutant infections to wildtype MGAS8232 using a two-way ANOVA with an uncorrected Fisher’s LSD test for multiple comparisons. **(C)** B6_HLA_ mice were euthanized, and lesions were excised 72 hours post-infection. Data points represent individual mice and bars represent the geometric mean. Dotted line represents the theoretical limit of detection. Statistical analysis comparing mutant infections to the wildtype MGAS8232 infection was done using a Kruskal-Wallis one-way ANOVA test and an uncorrected Dunn’s test for multiple comparisons. **(D)** Representative skin lesion images from B6_HLA_ mice 72 hours following skin challenge (* p < 0.05; ** p < 0.01; *** p < 0.001; **** p < 0.0001).

Although *S*. *pyogenes* primarily causes nasopharyngeal infection, it also remains a major cause of skin infections worldwide [1]. Previous work has shown that both SLO and SLS significantly contribute to *S*. *pyogenes* skin infection by promoting both lesion pathology and bacterial burden [18,20–24]. To examine the role of the streptolysins during skin infection of B6_HLA_ mice, mice were intradermally infected with wildtype *S*. *pyogenes* or the streptolysin-deficient strains. Briefly, mice were shaved and treated with hair-removal cream and then injected with 2.5×10^7^ CFU of bacteria in each flank, and weight and lesion sizes were measured daily for 72 hours. Mice infected with *S*. *pyogenes* Δ*sag* exhibited significantly less weight loss compared to mice infected with wildtype *S*. *pyogenes* at 24, 48, and 72 hours post-infection (**Fig 3A**). Additionally, mice infected with *S*. *pyogenes* Δ*sag* exhibited significantly smaller lesions at 24, 48, and 72 hours post-infection compared to mice infected with the wildtype strain (**Fig 3B and3D**). Although there was a significant reduction in lesion size of mice infected with *S*. *pyogenes* Δ*slo* 24 hours post-infection, lesion sizes were comparable to wild type infected lesions at 48 and 72 hours post-infection (**Fig 3B**). When examining bacterial burdens at 72 hours post-infection, there was a significant reduction in bacteria isolated from mice infected with *S*. *pyogenes* Δ*sag*, but not the Δ*slo* mutant strain, when compared to wildtype-infected mice (**Fig 3C**). Taken together, these data suggest that SLO is not a major contributor to *S*. *pyogenes* MGAS8232 skin infection in B6_HLA_ mice, while SLS significantly promotes both lesion pathology and bacterial burden during infection.

### Validation of streptolysin-deficient *S*. *pyogenes* strains

To confirm that the observed differences between the wildtype and streptolysin-deficient strains were a direct result of the loss of each respective toxin, the whole genomes for each *S*. *pyogenes* strain were sequenced and analyzed to check for any non-synonymous single nucleotide polymorphisms (SNPs) or other unexpected genetic alterations. We attempted to generate a complementation construct for the entire *sag* operon in *Escherichia coli*, however this was unsuccessful after multiple attempts and we hypothesize that the active *sag* operon is toxic in *E*. *coli*, as previously suggested [29]. *S*. *pyogenes* Δ*slo* and *S*. *pyogenes* Δ*sag* shared a SNP in the *ktrA* gene, which introduced a glutamic acid to lysine mutation (**S1 Table**). KtrA is the cytosolic portion of a potassium ion importer that regulates the transport capacity of the KtrB ion channel [30]. This SNP was found in both the Δ*slo* and Δ*sag S*. *pyogenes* strains, and since the *S*. *pyogenes* Δ*slo* strain is not attenuated in virulence, it’s unlikely contributing to any observed phenotype of *S*. *pyogenes* Δ*sag*. *S*. *pyogenes* Δ*sag* did have one additional SNP resulting in a valine to isoleucine substitution in the *lplA* gene, which encodes a putative lipoate protein ligase, and *S*. *pyogenes* Δ*slo* had an additional SNP in a pitrilysin family protein resulting in a frameshift mutation (**S1 Table**). To confirm our *in vivo* infection phenotypes were strictly due to loss of SLS production, we generated an independent mutation of the *sagA* gene alone in the *S*. *pyogenes* MGAS8232 backgrounds, and the pDCerm plasmid was used for plasmid-based complementation of *sagA*. Similar to the entire *sag* operon deletion strain, mice infected with *S*. *pyogenes* Δ*sagA* demonstrated a 100-fold reduction in bacterial burden in the nasopharynx compared to wildtype-infected mice 48 hours post-infection (**S2A Fig**). When mice were infected with the *sagA*-complemented strain, there was partial complementation of the wildtype-phenotype (**S2A Fig**). This result can be explained by the incomplete restoration of hemolysis of the plasmid-based complementation strain as seen on blood agar (**S2B Fig**). Together we can conclude that the decreased virulence of *S*. *pyogenes* Δ*sag* in the nasopharyngeal infection model was due to the loss of the SLS toxin.

### Neutrophil depletion restores bacterial burden of *S*. *pyogenes* Δ*sag* during skin but not nasopharyngeal infection

**Fig 4:**
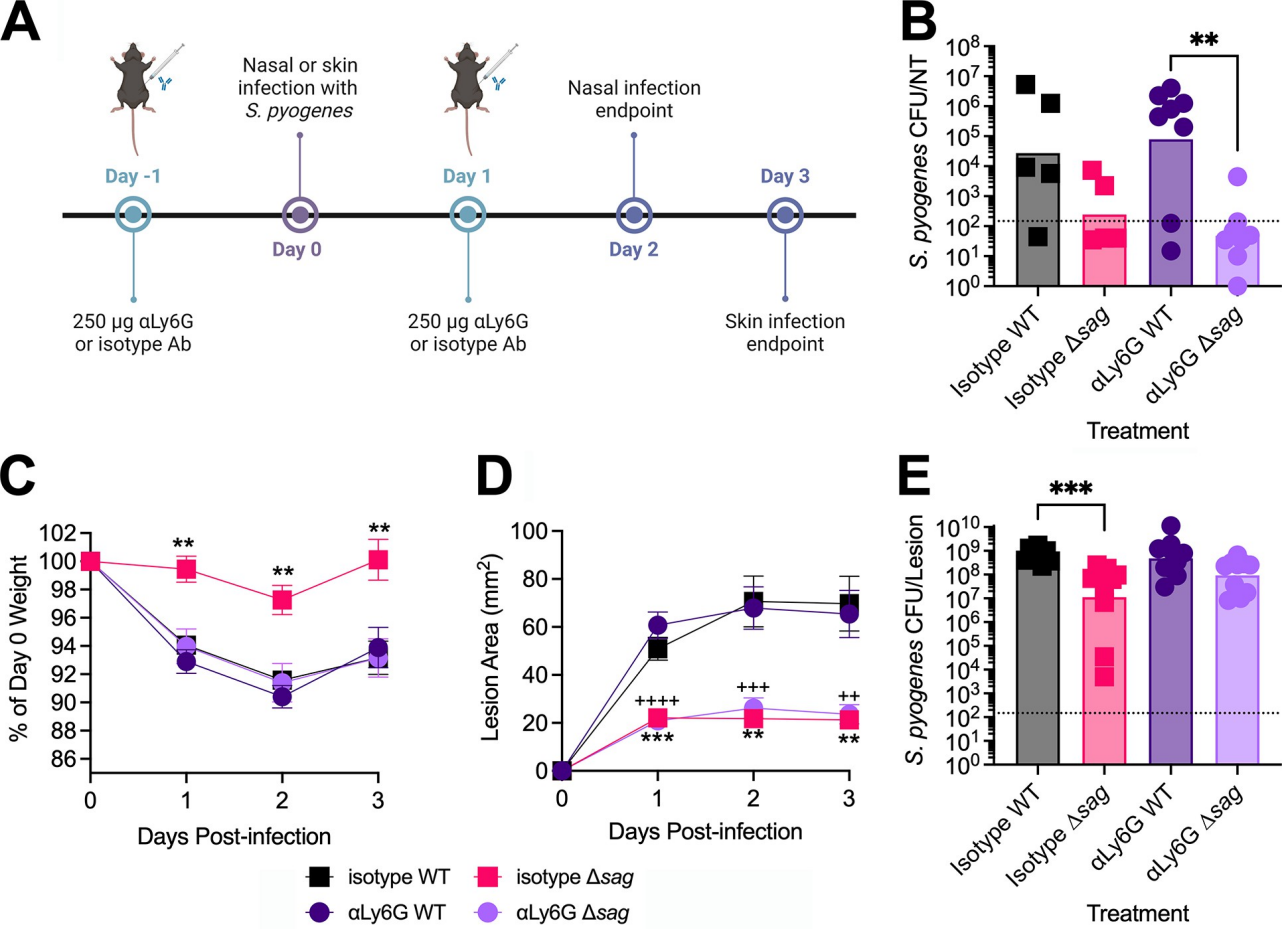
Neutrophil depletion restores *S*. *pyogenes* Δ*sag* bacterial burden during skin but not nasopharyngeal infection. **(A)** Timeline for neutrophil depletion during nasopharyngeal and skin infection. 8–12-week-old B6_HLA_ mice received intraperitoneal injections containing 250 μg of αLy6G antibody or the isotype control 24 hours before infection and 24 hours post-nasopharyngeal infection for a total of 500 ug/mouse. Created with BioRender.com**. (B)** Bacterial load in the nasal turbinates following neutrophil depletion. B6_HLA_ mice were infected with *S*. *pyogenes* and euthanized 48 hours post-infection. **(C)** Weights are presented as a percent of weight on day 0. **(D)** Lesions were measured using calipers at 24, 48, and 72 hours post-infection, and lesion area was calculated using two measurements perpendicular to each other. Data points represent mean +/- SE. **(E)** Data points represent individual mice and bars represent geometric means. **(B, E)** Statistical analysis was done comparing the wildtype and Δ*sag* infections for each antibody treatment and comparing the isotype and αLy6G antibody treatments for each *S*. *pyogenes* strain using a Kruskal-Wallis one-way ANOVA test and an uncorrected Dunn’s test for multiple comparisons. **(C, D)** Significance was determined by comparing wildtype MGAS8232 and Δ*sag* infections for isotype (*) or αLy6G (+) treatments using a two-way ANOVA with an uncorrected Fisher’s LSD test for multiple comparisons. Dotted line represents the theoretical limit of detection (**^/++^ p < 0.01; ***^/+++^ p < 0.001; ^++++^ p < 0.0001).

Previous studies have highlighted the ability of SLS to target and affect the migration of neutrophils during infection by *S*. *pyogenes*. In both a zebrafish model of myositis and a murine model of invasive skin infection, SLS-deficient *S*. *pyogenes* exhibited increased neutrophil influx to the site of infection [24,31]. Recently, we have shown that neutrophils are present during experimental nasopharyngeal infection of B6_HLA_ mice, and that capsule-deficient *S*. *pyogenes* exhibits an increased susceptibility to neutrophil-mediated killing [32]. Thus, we decided to explore whether *S*. *pyogenes* Δ*sag* exhibits a reduction in nasopharyngeal infection due to an increased susceptibility to neutrophil-mediated killing. Using a previously established protocol [32], mice were rendered neutropenic by administration of 250 μg of an αLy6G antibody, or an isotype control antibody, 24 hours prior to either nasopharyngeal or skin infection, and then given another dose of antibody 24 hours post-infection to ensure neutrophils remained depleted after infection (**Fig 4A**). Following nasopharyngeal infection, we found that SLS-deficient *S*. *pyogenes* was unable to infect neutrophil-depleted mice at comparable levels to the wildtype bacteria (**Fig 4B**). Although neutrophil depletion did not increase wildtype *S*. *pyogenes* burden in the nasal turbinates, there was an increased recovery observed in the lungs of neutropenic mice (**S3 Fig**). Mice given the isotype antibody and that were infected with *S*. *pyogenes* Δ*sag* exhibited significantly less weight loss throughout the course of infection compared to all other treatment groups (**Fig 4C**). However, throughout the course of skin infection, SLS-deficient *S*. *pyogenes* caused significantly smaller lesions compared the wildtype bacteria, independent of the presence or absence of neutrophils (**Fig 4D**). Expectedly, in control isotype-treated mice, a significant reduction in *S*. *pyogenes* Δ*sag* infection burden compared to wildtype-infected lesions was observed. While there was not a significant difference between the isotype or αLy6G treated mice infected with the Δ*sag* strain, neutrophil-depleted mice infected with SLS-deficient *S*. *pyogenes* exhibited an increased bacterial burden comparable to the wildtype strain (**Fig 4E**). Together, these data demonstrate that while SLS is not promoting nasopharyngeal infection through the disruption of neutrophil-mediated killing, it is, at least in part, contributing to skin infection by protecting the bacteria from neutrophils.

### Nasal infections with streptolysin-deficient *S*. *pyogenes* exhibit reduced macrophage influx and inflammation

**Fig 5:**
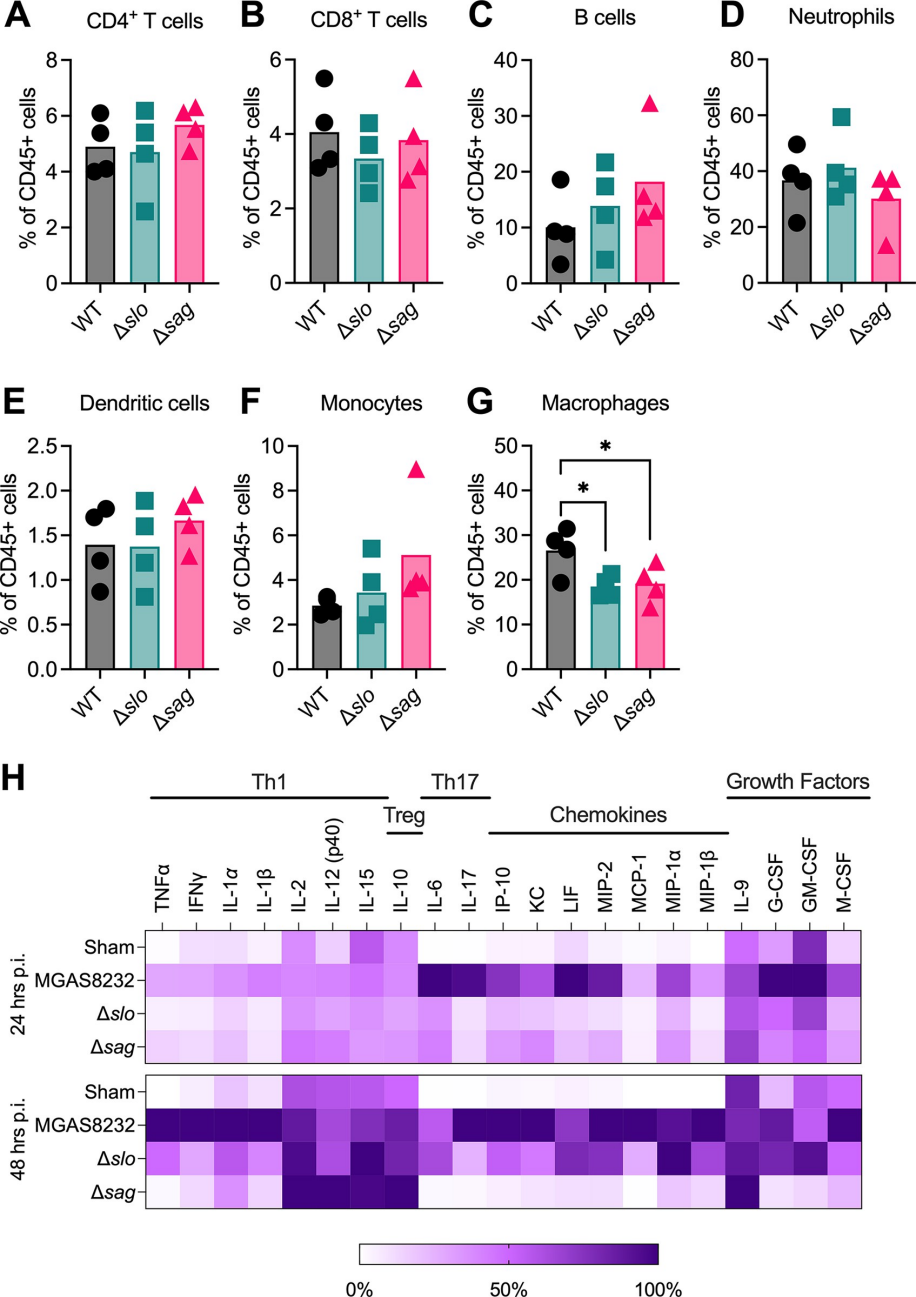
Immune cell and cytokine profiles from nasal turbinates of HLA-transgenic mice post *S*. ***pyogenes* infection.** B6_HLA_ mice were intranasally inoculated with ~1 × 10^8^ CFU of *S*. *pyogenes* MGAS8232 or the Δ*slo* or Δ*sag* mutant strains. Mice were euthanized **(A-H)** 24 hours or **(H)** 48 hours post-inoculation and **(A-G)** nasal homogenates were analyzed using flow cytometry or **(H)** nasal homogenate supernatants were sent for a cytokine array. **(H)** Data represents the mean cytokine concentration as a percent of the highest concentration for each cytokine (n ≥ 5). Statistical analysis was done comparing the mutant infections to the wildtype MGAS8232 infection in the nasal turbinates using a Kruskal-Wallis one-way ANOVA test and an uncorrected Dunn’s test for multiple comparisons (* p < 0.05; ** p < 0.01).

Next, we set out to further explore potential mechanisms by which SLS promotes nasopharyngeal infection. In addition to neutrophils, it has been shown that SLS can target and disrupt the function of various immune cells including macrophages [12,33–35]. To investigate the composition of immune cells present during nasopharyngeal infection, immune cell populations were assessed by flow cytometry from nasal tissues 24 hours post-infection. This time point was chosen due to the similar bacterial burden between the wildtype and streptolysin-deficient *S*. *pyogenes* strains (**Fig 2A**). When examining the immune cell composition in the nasal turbinates, we found no significant differences between the relative abundance of CD4^+^ T cells, CD8^+^ T cells, B cells, neutrophils, dendritic cells, or monocytes (**Fig 5A–5F**). Surprisingly, there was a significant difference in the relative abundance of macrophages, where mice infected with wildtype *S*. *pyogenes* had an increased relative abundance of macrophages compared to the streptolysin-deficient nasal infections (**Fig 5F**). Since streptolysin-deficient infections did not result in an increased abundance of any specific immune cell, the streptolysins are likely not targeting any one immune cell population to promote infection. Rather, wildtype *S*. *pyogenes* might be causing higher levels of inflammation, leading to an increase in macrophage abundance or activation in the nasal turbinates.

To assess the degree of inflammation in infected nasal turbinates, a cytokine array was conducted on nasal homogenate supernatants from infected mice 24 or 48 hours post-nasal infection. Unsurprisingly, mice infected with wildtype *S*. *pyogenes* exhibited a much stronger inflammatory signature compared to uninfected mice at both 24 and 48 hours post-infection (**Figs 5H and S4**). Interestingly, both streptolysin-deficient infections exhibited less inflammation 24 hours post-infection; however, only infection with the SLS-deficient strain resulted in an inflammatory signature comparable to the uninfected mice 48 hours post-infection (**Fig 5H**). While many cytokines are responsible for macrophage activation and chemotaxis, there are notable reductions in the macrophage chemokines MCP-1, MIP-1α and MIP-1β in streptolysin-deficient infections 24 hours post-infection. However, this reduction is only maintained in the SLS-deficient infections at 48 hours post-infection (**Fig 5H**). Overall, the presence of SLO or SLS results in an increased inflammation signature early in the infection timeline, while SLS promotes a sustained inflammatory response 48 hours post-infection.

### SLS promotes the destruction of the nasal epithelial barrier

**Fig 6:**
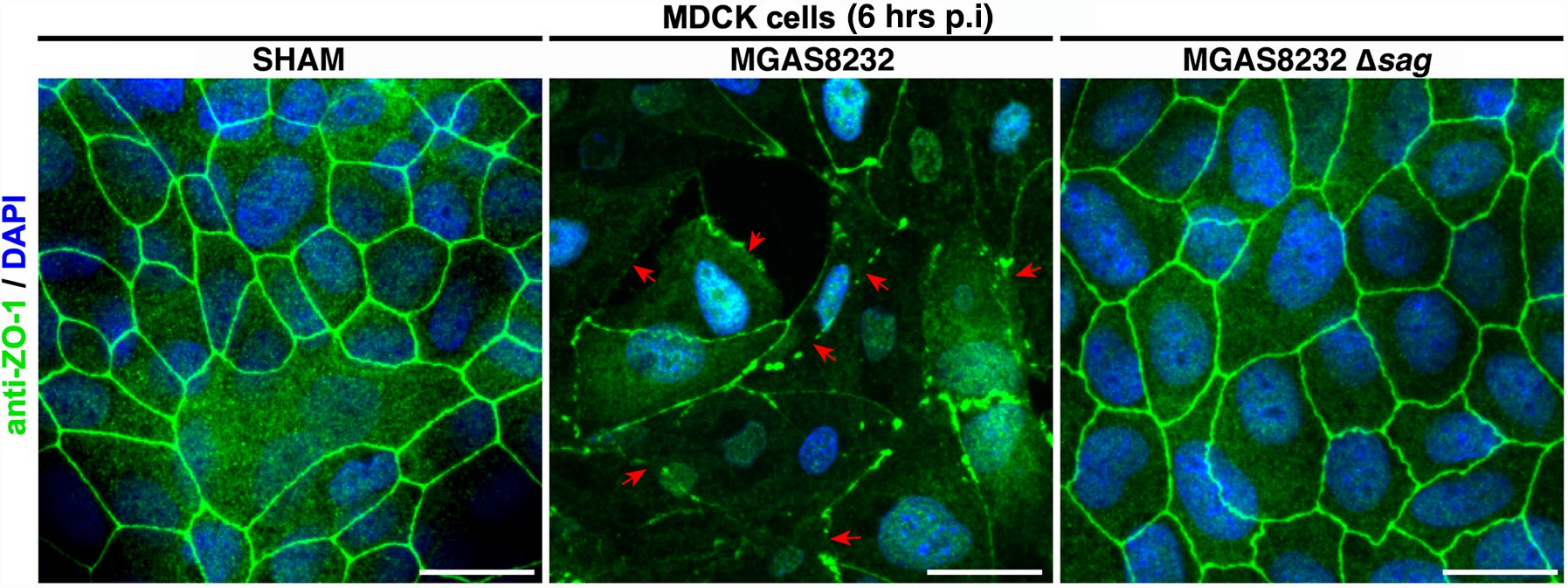
SLS contributes to the destruction of cell-cell junctions in MDCK cells. MDCK cells were grown to 100% confluency in DMEM + FBS and then infected with wildtype or Δ*sag S*. *pyogenes* MGAS8232 strains using an MOI of 100. Uninfected cells were used as a no treatment control (SHAM). At 6 hours post-infection, cells were fixed with 4% paraformaldehyde. For immunostaining, cells were stained with a primary rabbit anti-ZO-1 antibody, and then a secondary antibody using donkey anti-rabbit IgG Alexafluor488. Cells were also stained for 10 minutes with DAPI (10 μg/mL) and mounted onto glass slides using ProLong Diamond Antifade Mountant. Images were acquired by laser scanning confocal fluorescence microscopy using a Zeiss LSM 880 with Fast AiryScan. Representative images of ZO-1/DAPI stained MDCK cells were acquired, from multiple fields of view, as individual z-stacks each comprised of 10 slices. The images shown are z-projections of all 10 slices and show the sum of the fluorescence for each channel. Red arrows point to representative areas with aberrant ZO-1 staining. Scale bar is 20 microns.

In addition to immune cells, SLS can target epithelial cells and disrupt the epithelial barrier *in vitro* [17]. To begin to examine the role of SLS on epithelial barrier integrity, MDCK cells were infected with wildtype *S*. *pyogenes* or the Δ*sag* mutant and cell-cell junctions were analyzed through the staining of the tight junction protein ZO-1. We found that wildtype-infected cells exhibited a pattern of ZO-1 staining that was discontinuous, punctate, or non-existent (**Fig 6**). However, both uninfected cells (SHAM) and cells infected with the Δ*sag* strain, exhibited consistent staining of ZO-1 at the cell-cell interfaces, suggesting that SLS is contributing to the destruction of cell junctions *in vitro* (**Fig 6**).

**Fig 7:**
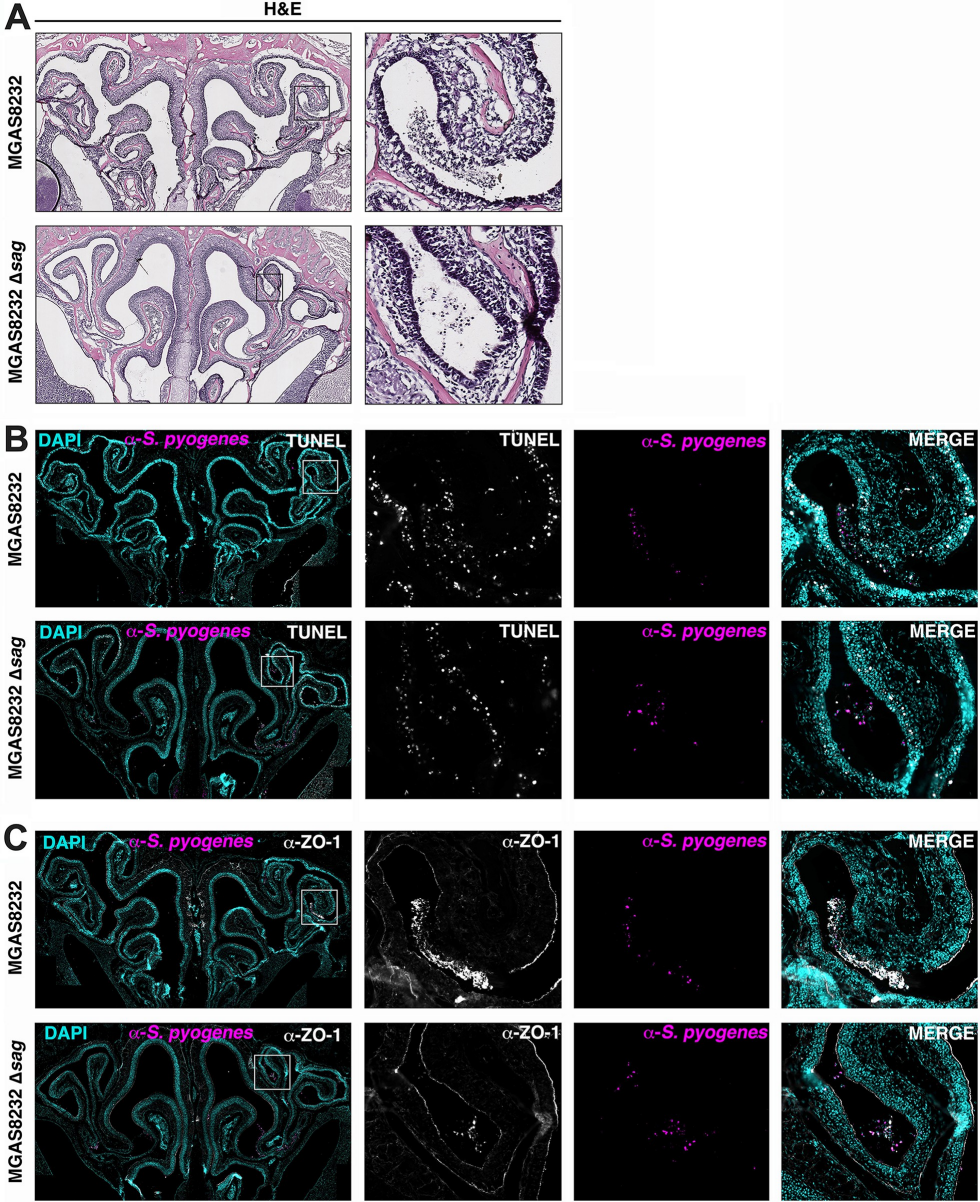
SLS contributes to nasal epithelial barrier destruction. B6_HLA_ mice were intranasally inoculated with ~1 × 10^8^ CFU of *S*. *pyogenes* MGAS8232 wild-type or Δ*sag*. Mice were euthanized 24 hours post-infection and heads were fixed in PLP fixation buffer and decalcified with EDTA. Tissues were embedded in OCT, flash frozen, and then sectioned using a cryostat. **(A)** Infected nasal turbinates stained with H & E. Images captured at 40X magnification. **(B)** Nasal turbinates stained with DAPI (teal), fluorescent TUNEL stain for apoptotic and necrotic cells (white) and immunofluorescent *S*. *pyogenes* stain (pink). **(C)** Epithelial barrier staining of nasal turbinates using DAPI (teal), immunofluorescent ZO-1 (white) and *S*. *pyogenes* (pink) staining. Boxes indicate location of zoomed-in images. **(B, C)** Whole images captured at 20X magnification and zoomed-in images captured at 40X magnification.

To explore whether SLS is causing nasal epithelial damage *in vivo*, mice were infected with wildtype *S*. *pyogenes* or the Δ*sag* mutant, and immunohistological analyses were done on the nasal turbinates of infected mice 24 hours post-infection, prior to the clearance of the Δ*sag* mutant in the nasal turbinates. H & E-stained nasal sections revealed that wildtype-infected mice exhibited increased epithelial damage indicated by an increase in cellular debris at the site of infection compared to Δ*sag*-infected mice (**Fig 7A**). To assess whether there was a difference in epithelial cell death, nasal sections were stained using a TUNEL stain to detect DNA breaks characteristic of apoptotic and necrotic cells. Compared to wildtype-infected mice, the SLS-deficient infections exhibited a slight decrease in TUNEL positive cells at the site of infection (**Fig 7B**). However, when sections were analyzed as a whole, there were no noticeable differences in TUNEL positive cells between the wildtype and mutant infection (**Fig 7B**). Next, we investigated the ability of SLS to specifically disrupt the epithelial barrier through the staining of the tight junction protein ZO-1. We found that at the localized site of infection, wildtype-infected mice exhibited a marked disruption of the epithelial barrier demonstrated by strong ZO-1 staining in the airway lumen, outside of the epithelial barrier (**Fig 7C**). In contrast, the Δ*sag*-infected mice have continuous ZO-1 staining along the epithelial barrier at the site of infection with minimal staining in the lumen (**Fig 7C**). Sections from sham-inoculated mice were also stained with H & E, TUNEL and ZO-1 with no notable damage to the nasal tissue present (**S5 Fig**). Together, these results provide evidence that SLS is promoting the destruction of the nasal epithelial barrier.

## Discussion

While *S*. *pyogenes* is responsible for over 700 million cases of infection each year, the vast majority of infections present as pharyngitis, and *S*. *pyogenes* is armed with an arsenal of secreted virulence factors that allow the bacteria to infect its human host [36,37]. In the context of pharyngitis, we previously found that the superantigen SpeA is essential for experimental nasopharyngeal infection by *S*. *pyogenes* through the induction of an inflammatory response [26]. Additionally, the secreted cysteine protease SpeB can contribute to nasopharyngeal infection in mice through the cleavage of IL-1β, also promoting inflammation and the clearance of endogenous microbiota [38]. Furthermore, in a study that also utilized an HLA-transgenic murine model of infection, the combined deletion of the superantigen SpeC and the DNAse SpdI resulted in a decrease in bacterial burden in the nasopharynx of mice [25]. Herein, we demonstrate that SLS is another secreted virulence factor that significantly contributes to both nasopharyngeal and skin infection in HLA-transgenic murine models of infection, and we provide evidence that suggests SLS is targeting the nasal epithelial barrier to promote nasopharyngeal infection.

Although the streptolysins have not been extensively studied in the context of pharyngitis, SLO has been implicated in nasopharyngeal infection, with an SLO-deletion mutant exhibiting over a 10-fold reduction in bacterial burden in the nasopharynx of HLA-transgenic mice [25]. While we did not find a significant contribution of SLO to nasopharyngeal infection with *S*. *pyogenes* MGAS8232, there are notable differences between the strains used. Using the *S*. *pyogenes* HKU16 strain, Brouwer, S *et al*. highlighted the role of SLO pores in reducing the extracellular environment through the release of intracellular GSH, resulting in the thiol-activation of the superantigen SSA in this reducing environment. Therefore, SLO may be indirectly promoting infection through the activation of SSA, or through the combined cytotoxic effects of SLO and SSA. However, *S*. *pyogenes* MGAS8232 relies on the superantigen SpeA for nasopharyngeal infection of transgenic mice and does not encode the streptococcal superantigen SSA [26,28]. Additionally, *S*. *pyogenes* HKU16 encodes an active form of the streptococcal NADase in the *slo* operon, while the NADase in *S*. *pyogenes* MGAS8232 contains amino acid substitutions at two key residues rendering it inactive [28,39–41]. Studies have also suggested that the entry of the NADase into host cells is reliant on the formation of the SLO pore [42,43]. Therefore, the observed loss of fitness of the *S*. *pyogenes* HKU16 Δ*slo* could, in part, be attributed to a loss of NADase entry into host cells. Recently, a study found that SLO and SLS are differentially expressed depending on the primary carbon source being metabolized, demonstrating that differences in carbon source utilization between strains can further influence toxin expression [44]. Although *S*. *pyogenes* MGAS8232 does not express an active NADase, the study analyzing NADase activity also found that 48.6% (55/113) of *S*. *pyogenes* strains examined had a mutation abolishing NADase activity, highlighting the frequency of this mutation among *S*. *pyogenes* strains [41]. Together, these strain-specific differences highlight the heterogeneity of virulence factor expression and regulation among *S*. *pyogenes* strains and environmental conditions.

Surprisingly, outside of a modest difference in lesion size 24 hours post-infection, SLO did not significantly contribute to skin infection (**Fig 3**). While previous studies have used conventional mice to assess the role of SLO and SLS during skin infection, our study used HLA-transgenic mice. These mice are susceptible to bacterial superantigens, which manipulate the host immune response through the hyper-activation of T cells. Recently, the superantigen SpeC has been shown to contribute to skin infection in HLA-transgenic mice [45]. Therefore, using infection models where the superantigens are active may result in an altered immunological environment compared to that of conventional mice, and could be more replicative of a natural human infection. Unlike SLO, our finding that SLS significantly contributes to skin infection was expected, as multiple studies using different strains of *S*. *pyogenes* have implicated SLS in lesion formation and bacterial burden [22,23]. Furthermore, SLS has recently been implicated in the disease progression of necrotizing fasciitis, where SLS directly activated nociceptor neurons to cause pain during infection, resulting in the release of a neuropeptide responsible for inhibiting the recruitment of neutrophils and opsonophagocytic killing [46]. This concurs with our study demonstrating that neutrophil depletion restores the infection burden of an SLS mutant strain during skin infection (**Fig 4E**).

Recently, we demonstrated that neutrophils are present during our experimental nasopharyngeal infection, and that the hyaluronic acid capsule is critical to avoid neutrophil-mediated killing [32]. Here, when mice were depleted of neutrophils, the bacterial burden of the SLS-deficient *S*. *pyogenes* nasopharyngeal infection was unaffected (**Fig 4**). We also noted that neutrophil-deplete mice infected with the wildtype bacteria was also unaffected yet had increased amounts of bacteria present in their lungs (**S3 Fig**), which concurs with a study highlighting the role of neutrophils in preventing streptococcal pneumonia in mice [47]. Although neutrophil depletion did not restore bacterial burden in the nasopharynx, bacterial burden was recovered in a streptococcal skin infection (**Fig 4E**), which was expected given that the lack of SLS is associated with increased neutrophils during skin infection in mice [24].

To determine if SLO or SLS were selectively targeting a specific immune cell type during nasopharyngeal infection, we assessed different cell populations by flow cytometry 24 hours post-infection. If a specific cell type was targeted by the toxins during nasal infection, we would expect that cell type to be enriched in the respective SLO- or SLS-deficient infections. However, we found a significantly higher proportion of macrophages in wildtype-infected mice compared to the streptolysin-deficient infections (**Fig 5F**). We also found that mice infected with the wildtype *S*. *pyogenes* strain exhibited a stronger inflammatory signature compared to the streptolysin-deficient strains 24 hours post-infection (**Fig 5H**). As cytolytic toxins, the inflammatory nature of SLO and SLS is expected. For example, keratinocytes infected with SLO-deficient mutants of *S*. *pyogenes* exhibited reduced production of the proinflammatory cytokines IL-1β, IL-6 and IL-8 compared to wildtype *S*. *pyogenes*, and SLS can induce the production of IL-1β during skin infection [10,48]. Therefore, it’s possible the increased proportion of macrophages in the nasopharynx is due to an increased influx or activation of macrophages in response to the inflammation caused by the cytolysins. However, due to limited sample size (n = 4), this remains to be fully explored.

Since SLS was not selectively targeting a specific immune cell type, we hypothesized that SLS-induced inflammation was from epithelial cell damage. SLS has been shown to work in conjunction with the host protease calpain to facilitate the destruction of intracellular junction proteins, allowing for paracellular invasion of the bacteria *in vitro* [17]. Therefore, to examine epithelial barrier integrity, we first examined the ability of the wildtype and Δ*sag S*. *pyogenes* strains to cause damage to the cell-cell junctions of MDCK cells. MDCK cells were chosen as they form densely packed monolayers and are known to express cell-cell junction proteins [49]. As expected, we found that wildtype-infected cells exhibited damaged cell-cell junctions indicated by the disruption of ZO-1 staining that was absent in cells infected with the SLS-deficient strain (**Fig 6**).

Additionally, at the localized site of infection *in vivo*, we found a marked disruption of the epithelial barrier of wildtype-infected mice that was not present in mice infected with the SLS-deficient infection (**Fig 7C**). To avoid mucociliary clearance by the host, disruption of the epithelial barrier may expose host molecules such as collagen IV and fibronectin, which could improve adhesion of *S*. *pyogenes* to host cells [50–52]. Additionally, the release of nutrients from epithelial cells caused by barrier destruction may also promote the establishment of nasopharyngeal infection. Overall, we have demonstrated that SLS is essential for the establishment of nasopharyngeal infection and significantly contributes to skin infection in HLA-transgenic mice. Furthermore, we provide evidence that SLS promotes nasopharyngeal infection through the destruction of the epithelial barrier. Given the importance of SLS in the two most common manifestations of *S*. *pyogenes* disease, this work has the potential to guide the development of therapies to prevent and treat *S*. *pyogenes* infections by targeting SLS-induced pathogenesis.

## Methods

### Ethics statement

Human venous blood was taken from healthy volunteer donors, following written consent, in accordance with human subject protocol 110859. The full study protocol was approved by the London Health Sciences Centre Research Ethics Board (University of Western Ontario, London, ON, Canada).

All animal experiments were conducted in accordance with the Canadian Council on Animal Care Guide to the Care and Use of Experimental Animals. Animal protocol (2020–041) was approved by the Animal Use Subcommittee at the University of Western Ontario. These mice were bred in a barrier facility at the University of Western Ontario and were routinely genotyped for the appropriate transgenes.

### Bacterial strains

A complete list of strains and plasmids used in this study can be found in **Table 1**. All recombinant plasmids were built with standard molecular procedures using *E*. *coli* XL1-blue as the cloning host [53]. *E*. *coli* strains were grown in Luria-Bertani (LB) broth with the appropriate antibiotic. *S*. *pyogenes* MGAS8232 strains were grown in Todd Hewitt media supplemented with 1% (w/v) yeast extract (THY; BD Biosciences, USA) at 37°C. Genomic deletions were made using the pG^+^host5 Gram-positive system [54]. Briefly, deletion constructs were generated by amplification of 500–1000 bp of DNA on either side of the operon of interest (primers listed in **S2 Table**) and cloned into pG^+^host5. Flanking DNA regions included the first and last 3 codons of the operon to generate a precise, markerless and in-frame deletion. Recombinant plasmids were then electroporated (Bio-Rad Gene Pulser XCell) into electrocompetent *S*. *pyogenes* MGAS8232 cells. Single crossover integrations were selected at 40°C under erythromycin (1 μg/ml) selection, and then grown without antibiotics at 30°C to be screened for a loss of erythromycin resistance. Double crossover gene disruptions were then confirmed by PCR, and PCR products were sequenced. Sanger sequencing of PCR products and plasmids inserts was done using sequencing primers listed in **S2 Table** and was done by the John P. Robarts Research Institute sequencing facility at the University of Western Ontario in London, Ontario, Canada.

**Table 1:** Bacterial strains and plasmids used in this study.

Bacterial Strain	Description	Source
*Escherichia coli*		
XL1-Blue	Cloning host	Stratagene
*Streptococcus pyogenes*		
MGAS8232	M18 serotype isolated from a patient with acute rheumatic fever (GenBank accession: NC_003485.1)	[28]
MGAS8232 Δ*slo*	MGAS8232 containing an in-frame deletion of the *slo* operon	This study
MGAS8232 Δ*sag*	MGAS8232 containing an in-frame deletion of the *sag* operon	This study
MGAS8232 Δ*sagA*	MGAS8232 containing an in-frame deletion of the *sagA* gene	This study
**Plasmids**		
pG^+^host5	Temperature-sensitive Gram-positive/*E*. *coli* shuttle vector, Erythromycin^r^	[54]
pG^+^host5:: Δ*sag*	Recombinant plasmid containing *sag* deletion construct	This study
pG^+^host5:: Δ*slo*	Recombinant plasmid containing *slo* deletion construct	This study
pG^+^host5:: Δ*sagA*	Recombinant plasmid containing *sagA* deletion construct	This study
pDCerm::*sagA*	pDCerm expressing sagA gene for plasmid-based complementation	[61]

All strains were tested for growth defects using a Synergy H4 Hybrid Reader (BioTek Instruments, USA) for growth assays. Furthermore, differences in global protein expression were assessed using a trichloroacetic acid precipitation of culture supernatants and SDS-PAGE. Lastly, an anti-SpeA western blot was done to ensure expression of this key virulence factor was not altered between the mutant strains [26]. Purified SpeA was used to generate polyclonal rabbit antibodies from a commercial source (ProSci Incorporated, USA) and were detected using an anti-rabbit IR800 secondary antibody (LI-COR Biosciences, USA). To assess hemolysis of *S*. *pyogenes* strains, bacteria were grown at 37°C overnight on tryptic soy agar supplemented with 5% sheep’s blood.

### Whole genome sequencing

For whole genome sequencing, genomic DNA preparations from *S*. *pyogenes* MGAS8232 wildtype, Δ*slo*, Δ*sag*, and Δ*sagA* strains were sent for paired end Illumina sequencing at the John P. Robarts Research Institute sequencing facility (University of Western Ontario, London, Ontario). Illumina short-read sequence data were used to generate *de novo* assemblies using SPAdes v3.15 [55], which were annotated using Prokka v1.12 [56]. These assemblies have been deposited at NCBI. Any sequence differences between the strains were determined using Snippy v4.1 (https://github.com/tseemann/snippy), where strains were the Δ*slo*, Δ*sag* strains were compared to *S*. *pyogenes* MGAS8232 wildtype strain to determine single nucleotide polymorphisms.

### Whole blood survival assay

To examine bacterial survival in whole human blood, a Lancefield bactericidal assay was performed as previously described, with some modifications [57]. Briefly, blood from a human donor was inoculated with 1000 CFU ml^-1^ of bacteria. Samples were incubated in 1 ml aliquots at 37°C with shaking. At time points of 0, 30, 60, 90, 120, and 180 minutes, samples were serially diluted and plated on TSA with 5% sheep blood plates in triplicate to determine bacterial concentration.

### Hemolysis assay

Hemolysis assays were performed as previously described [58]. Briefly, culture supernatants were isolated from stationary phase cultures (OD_600_ 0.8 to 1.0) of *S*. *pyogenes*. Supernatants were reduced by adding 20 mM L-cysteine and incubating the mixture at room temperature for 10 minutes. A 5% (v/v) solution of human RBCs was made using blood from a human donor. RBCs were washed three times and resuspended in PBS (pH 7.3) and were treated with the reduced stationary phase culture supernatants from the bacterial strains at a ratio of 4:1. Samples were then incubated at 37°C for 60 minutes, then centrifuged at 500 × *g* to pellet the remaining cells. The A_540_ of the supernatants was measured to determine the amount of hemoglobin released. RBCs treated with 0.1% (v/v) Triton X-100 (Bio-Rad Laboratories, USA) were used as a positive control to measure percent lysis.

### *S*. *pyogenes* nasopharyngeal infection

8-12-week old HLA-expressing transgenic mice (B6_HLA_) were used to enhance nasopharyngeal infection by *S*. *pyogenes* [26]. Mice were infected following a previously established murine model of nasopharyngeal infection [59]. Briefly, freshly subcultured exponential phase *S*. *pyogenes* cells (OD_600_ 0.2 to 0.3) were washed twice and suspended in Hank’s Buffered Saline Solution (HBSS). B6_HLA_ mice were inoculated with 7.5 μl in each nostril (a total of 1 ± 0.4 ×10^8^ CFU) under Forane (isoflurane, USP) inhalation anesthetic (Baxter Corporation, Canada). Mice were sacrificed 24 or 48 hours post-infection, and the nasal turbinates were removed. Nasal tissue was homogenized, serially diluted, and plated in duplicate on TSA with 5% sheep blood.

### *S*. *pyogenes* skin infection

8-12-week-old B6_HLA_ mice were shaved, and the remaining fur was removed using commercial hair removal cream. Mice were injected subcutaneously 24 hours later with 5×10^7^ CFU of bacteria in 100 μl, with 2.5×10^7^ (50 μl) injected into each flank. The area of each lesion was measured daily by using calipers to take two perpendicular measurements, and the weight of each mouse was measured daily. Mice were sacrificed 72 hours post-infection, and lesions were excised, homogenized, and plated to determine the number of bacteria per lesion.

### Mouse cytokine arrays

Cytokine concentrations were determined from nasal homogenates isolated from mice treated with HBSS (SHAM), *S*. *pyogenes* MGAS8232, *S*. *pyogenes* MGAS8232 Δ*slo*, or *S*. *pyogenes* Δ*sag* at either 24 or 48 hours post-infection in B6_HLA_ mice. Multiplex bead arrays were performed using the Mouse Cytokine 32-plex Discovery Array (Eve Technologies).

### Neutrophil depletion

Neutrophils were depleted *in vivo* by intraperitoneally injecting 8–12 week old mice with 250 μg mAb αLy6G clone 1A8 (Cedarlane) 24 hours before and 24 hours following nasopharyngeal and skin infections. Control mice received rat IgG2a clone 2A3 (Cedarlane). Depletion of circulating neutrophils using this protocol has been confirmed in a previous study by flow cytometry of Ly6G^+^ expressing populations in blood and the nasopharynx [32]. Bacterial burden in nasal turbinates was examined at both 48 hours following infection.

### Flow cytometry

To assess immune cell populations during nasopharyngeal infection, flow cytometry was performed on nasal turbinates and blood. Murine nasal turbinates were isolated as previously described [59] and collected in R10 media. Nasal turbinates were treated with 0.3 mg ml^-1^ collagenase D (Sigma-Aldrich) in R10 media at 37°C for 30 minutes and then pushed through a 0.7-μm cell strainer. The single-cell suspension was then treated with ACK lysis buffer (Gibco) and washed with PBS containing 2% FBS. Blood was collected from mice via cardiac puncture. Following isolation, blood was treated with ACK lysis buffer (Gibco) and washed with PBS containing 2% FBS. Following isolation, both blood and nasal cells were stained and analysed as follows: Cell viability was first determined using Fixable Viability Dye eFluor506 (Thermo Fisher) and then subsequently stained with anti-CD45-BV421 (clone 30-F11, Biolegend), anti-CD19-BV711 (clone 1D3, BD), anti-CD4-PE-Cy5 (clone RM4-5, Thermo Fisher), anti-CD8a-PerCP (clone 53–6.7, Biolegend), anti-F4/80-A647 (clone BM8, Biolegend), anti-Ly6G-A700 (clone RB6-8C5, Biolegend), anti-Ly6C-PE (clone HK1.4, Biolegend), anti-CD11b-A488 (clone M1/70, Biolegend), and anti-CD11c-APC-Cy7 (clone HL3, BD). Cells were fixed overnight with 1% paraformaldehyde prior to analysis. Events were acquired using a LSR II (BD Biosciences), and data were analyzed using FlowJo v10.7.1 (TreeStar).

Cells were first gated for the singlet population and then subsequently gated for whether they were alive or dead. Single live cells were checked for the expression of CD45 to determine the immune cell pool. Cells were assessed for the expression of CD19 and classed as B cells. Cells were then assessed for the expression of CD4 or CD8 and classed as CD4^+^ or CD8^+^ T cells. Cells that were negative for CD19, CD4 and CD8 were gated for further analysis. Cells were then assessed for expression of F4/80 and Ly6G. F4/80 positive and Ly6G negative cells were classed as macrophages. Cells that were subsequently high for Ly6G but negative for F4/80 were assessed for CD11b and Ly6C expression and classed as neutrophils. F4/80 and Ly6G double negative cells were gated and assessed for CD11b and Ly6C expression, with double positive cells classed as monocytes. Ly6G and F4/80 double negative cells were also gated and assessed for CD11c and Ly6C expression, with CD11c^+^/Ly6C^-^ cells classed as dendritic cells. Data is presented as the percentage of live immune cells.

### Madin-Darby canine kidney (MDCK) cell culture and infection

MDCK cells were grown in Dulbecco’s Modification Eagle’s Medium containing 4.5 g L^-1^ glucose, L-glutamine, and sodium pyruvate, supplemented with 10% FBS (DMEM) at 37°C in 5% CO_2_. For cell culture experiments, MDCK cells were grown to confluency in 12 well plates (Falcon) on 18 mm (No.1) glass cover slips (Matsunami Glass). For cell culture infections, overnight bacterial cultures were subcultured 1:20 and grown for 5 hours to and OD_600_ of 0.8–1. Bacteria were washed twice in PBS, resuspended in DMEM with FBS, and added to MDCK cells at an MOI of 50 to 100. Immediately following infection, cell culture plates were centrifuged at 300 × *g* for 2 minutes, and then incubated for 6 hours. Following incubation, wells were washed with PBS and then fixed with 4% PFA for 20 minutes at room temperature. Cells were then washed with PBS containing 2 mM glycine to quench any residual PFA, and stored in PBS at 4°C.

### MDCK cell immunostaining and microscopy

For immunostaining, glass coverslips with PFA fixed MDCK cells were first permeabilized with 0.1% Triton X-100 diluted in PBS for 20 minutes at room temperature. Next, permeabilized cells were blocked with 8% (w/v) skim milk in PBS for at least 4 hours at 4°C. Following blocking, cells were then stained with primary rabbit anti-ZO-1 (ThermoFisher, 61–7300) diluted 1:100 in 0.8% skim milk and incubated for 1.5 hours at room temperature. After washing, permeabilized cells were stained with secondary antibody using donkey anti-rabbit IgG Alexafluor488 (ThermoFisher) that was diluted 1:1000 in 0.8% skim milk for ~45 minutes at room temperature. The cells were washed twice then stained for 10 minutes with DAPI (10 μg/mL) diluted in PBS and then washed again twice. Stained coverslips were mounted onto glass slides using ProLong Diamond Antifade Mountant (ThermoFisher) and dried at room temperature. Images were acquired by laser scanning confocal fluorescence microscopy using a Zeiss LSM 880 with Fast AiryScan. The microscope apparatus is comprised of a Zeiss Axio Observer microscope equipped with a Plan-Apochromat ×63/1.4 oil DIC M27 objective, a fully motorized stage and a Definite Focus 2. The microscope is equipped with the following lasers: argon (458 nm, 488 nm, 514), diode 405–30 (405 nm), DSPSS 561–10 (561 nm), and HeNe633 (633 nm), and an X-Cite LED (EXCELITAS Technologies) light source for epifluorescence. Signal detection makes use of 3 GaASP detectors and a T-PMT. The microscope is controlled by the Zen Black software (Zeiss). Excitation of Alexafluor 488 dye used the argon laser, and the diode 405 was used for DAPI excitation.

Representative images of ZO-1/DAPI stained MDCK cells were acquired, from multiple fields of view, as individual z-stacks each comprised of 10 slices. Post-image processing was done using the open-source platform FIJI [60]. Briefly, z stacks were projected, showing the sum of the fluorescence intensity for each channel, into a 2D image. Z projected images were then similarly contrast enhanced and cropped using FIJI.

### Histology

At 24 hours post-infection, mice were euthanized, and nasal turbinates were collected for histological analyses as previously described [59]. Tissues were fixed in periodate-lysine-paraformaldehyde (PLP) for 24 hours at 4°C and then treated with 14% EDTA buffer until the bones were decalcified, changing the EDTA buffer every 48 hours. Following fixation and decalcification, nasal turbinates were passed through sucrose gradients and flash frozen in OCT (TissueTek) media. Serial sections (7 μm) were cut using a cryostat.

### H & E staining

For H & E-stained sections, slides were air dried and dipped in Modified Mayer’s Hematoxylin (Fisher Scientific) for 10 minutes. Slides were then rinsed with cool distilled water and dipped in 1% Eosin (Fisher Scientific) 12 times. Slides were then rinsed in distilled water, then dipped in 50% ethanol 10 times and 70% ethanol 10 times before being equilibrated in 95% ethanol for 30 seconds and 100% ethanol for 1 minute. Slides were then dipped in xylene to remove excess water and mounted with Entellan mounting media (Sigma-Aldrich). Tiled images of whole nasal turbinate sections were collected using a Aperio AT2 scanner (Lecia Biosystems) at 40X magnification. Post-image processing was done using the open-source platform FIJI [60].

### Immunofluorescent staining

For epithelial barrier staining, slides were thawed at room temperature, washed with PBS, and blocked with Powerblock Universal Blocking Reagent (BioGene) for 30 minutes. Sections were then stained with the primary rabbit anti-ZO-1 (ThermoFisher, 61–7300) diluted 1:100 in 5% donkey serum and 0.1% Tween20 in PBS overnight at 4°C. Following overnight incubation, slides were washed with PBS and sections were then stained with the primary goat anti-*S*. *pyogenes* Group A Carbohydrate (Abcam, ab9191) diluted 1:100 in 5% donkey serum and 0.1% Tween20 in PBS for 1 hour at room temperature. For secondary antibody staining, donkey anti-goat IgG Alexafluor594 (ThermoFisher) and donkey anti-rabbit Alexafluor647 (ThermoFisher) were diluted 1:500 in 5% donkey serum and 0.1% Tween20 in PBS for 1 hour at room temperature. Sections were stained with DAPI (5 μg/ml) for 5 minutes and washed with 0.1% Tween20 in PBS. After staining, sections were mounted with ProLong Gold Antifade Reagent (Invitrogen). Tiled images of whole nasal turbinate sections were collected using a DM5500B fluorescence microscope (Leica Biosystems) at 20X and 40X magnification. Post-image processing was done using the open-source platform FIJI [60].

To stain apoptotic and necrotic cells, a TUNEL stain was done on sectioned tissues using the Click-iT Plus TUNEL Assay Kits for In Situ Apoptosis Detection (ThermoFisher Scientific). Staining was done following the manufacturer’s instructions with a few modifications. Since tissues were cryopreserved, tissues were permeabilized with 0.25% Triton in PBS for 15 minutes. *S*. *pyogenes* and DAPI staining was done as described above, beginning with blocking.

### Statistical analysis

All statistical analysis were done using the Prism9 software (GraphPad).

## Supporting information

S1 Fig *In vitro* analyses of streptolysin deletion mutants. **(A)** *S*. *pyogenes* MGAS8232 wildtype, Δ*slo*, or Δ*sag* strains grown in THY broth. **(B)** Trichloroacetic acid precipitation of overnight culture supernatants were collected and a Western blot against SpeA was done. Recombinant SpeA was used as a positive control.
(TIF)

S2 Fig Deletion of *sagA* alone results in a decrease in nasopharyngeal infection. **(A)** 8–12-week-old B6_HLA_ mice were nasally inoculated with ~1×10^8^ CFU of either the wildtype *S*. *pyogenes* MGAS8232, Δ*sagA*, or Δ*sagA* + *sagA* strain. Bacterial burden in the nasal turbinates was determined 48 hours post-inoculation. Data points represent individual mice and bars represent the geometric mean. Dotted line represents the limit of detection (10^2.17^). Statistical analysis was done using a Kruskal-Wallis test and an uncorrected Dunn’s test for multiple comparisons. **(B)** *S*. *pyogenes* MGAS8232 strains grown on TSA with 5% sheep’s blood (** p < 0.01).
(TIF)

S3 Fig Bacterial burden in the lungs following neutrophil depletion. **(A)** 8–12-week-old B6_HLA_ mice received intraperitoneal injections containing 250 μg of αLy6G antibody or the isotype control 24 hours before infection and 24 hours post-nasopharyngeal infection for a total of 500 μg/mouse. B6_HLA_ mice were infected with *S*. *pyogenes* and euthanized 48 hours post-infection. Data points represent individual mice and bars represent the geometric mean. Statistical analysis was done using a Kruskal-Wallis test and an uncorrected Dunn’s test for multiple comparisons.
(TIF)

S4 Fig Cytokine response in nasal turbinates of B6_HLA_ mice during streptococcal infection. Mice were inoculated with HBSS as sham control or infected intranasally with ~1×10^8^ CFU of *S*. *pyogenes* MGAS8232 wildtype, Δ*slo*, or Δ*sag* strains. Mice were euthanized 24 or 48 h post-infection and nasal turbinate homogenates were analyzed using a cytokine array **(A)** Th1-type cytokines **(B)** Th2-type cytokines **(C)** Treg cytokines **(D)** Th17 cytokines **(E)** chemokines or **(F)** growth factors. Data represents the mean ± SEM of nasal turbinate cytokine/chemokine concentrations (n ≥ 3 mice per group). Significance was determined by comparing groups to the wildtype infection at either 24- or 48-hours post-infection using a one-way ANOVA with an uncorrected Fisher’s LSD test (* p < 0.05; ** p < 0.01; *** p < 0.001; **** p < 0.0001).
(TIF)

S5 Fig Staining of sham-inoculated nasal turbinates. B6_HLA_ mice were intranasally inoculated with HBSS as a sham and euthanized 24 hours post-infection. Heads were fixed in PLP fixation buffer and decalcified with EDTA. Tissues were embedded in OCT, flash frozen, and then sectioned using a cryostat. **(A)** Nasal turbinates stained with H & E. Images captured at 40X magnification. **(B)** Nasal turbinates stained with DAPI (teal), fluorescent TUNEL stain for apoptotic and necrotic cells (white) and immunofluorescent *S*. *pyogenes* stain (pink). **(C)** Epithelial barrier staining of nasal turbinates using DAPI (teal), immunofluorescent ZO-1 (white) and *S*. *pyogenes* (pink) staining. **(B, C)** Images captured at 20X magnification.
(TIF)

S1 Table SNPs identified from genome wide comparison to *S*. *pyogenes* MGAS8232 wildtype strain. (DOCX)

S2 Table Primers used in this study. (DOCX)
