# Supplementary figures and images for: Streptolysin S is required for Streptococcus pyogenes nasopharyngeal and skin infection in HLA-transgenic mice
Source: PLoS Pathog. 2024 Mar 7;20(3):e1012072. doi: 10.1371/journal.ppat.1012072 (PMC10950238; doi:10.1371/journal.ppat.1012072)

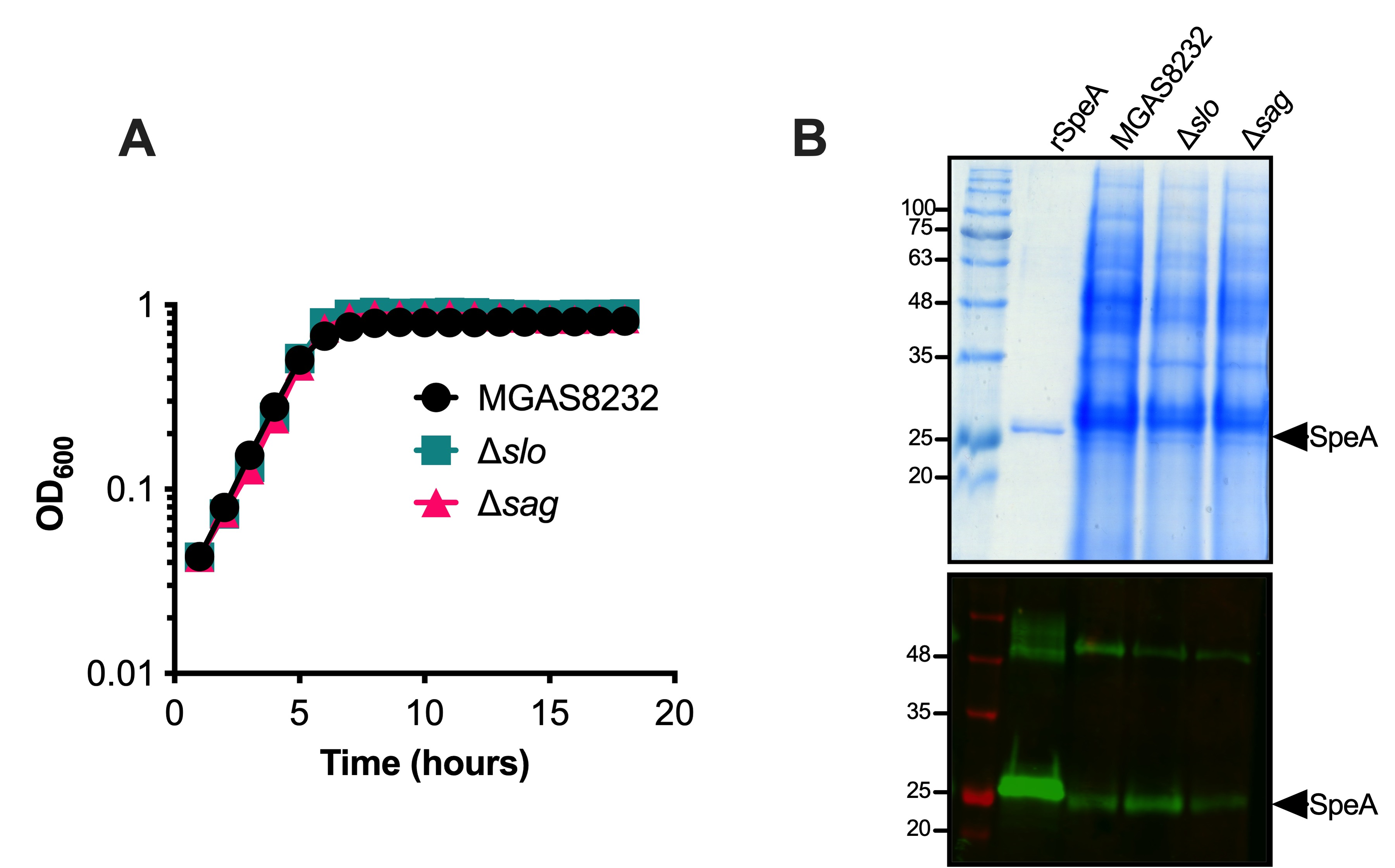

Supplement: S1 Fig — (A) S. pyogenes MGAS8232 wildtype, Δslo, or Δsag strains grown in THY broth. (B) Trichloroacetic acid precipitation of overnight culture supernatants were collected and a Western blot against SpeA was done. Recombinant SpeA was used as a positive control. (TIF) [file ppat.1012072.s001.tif]

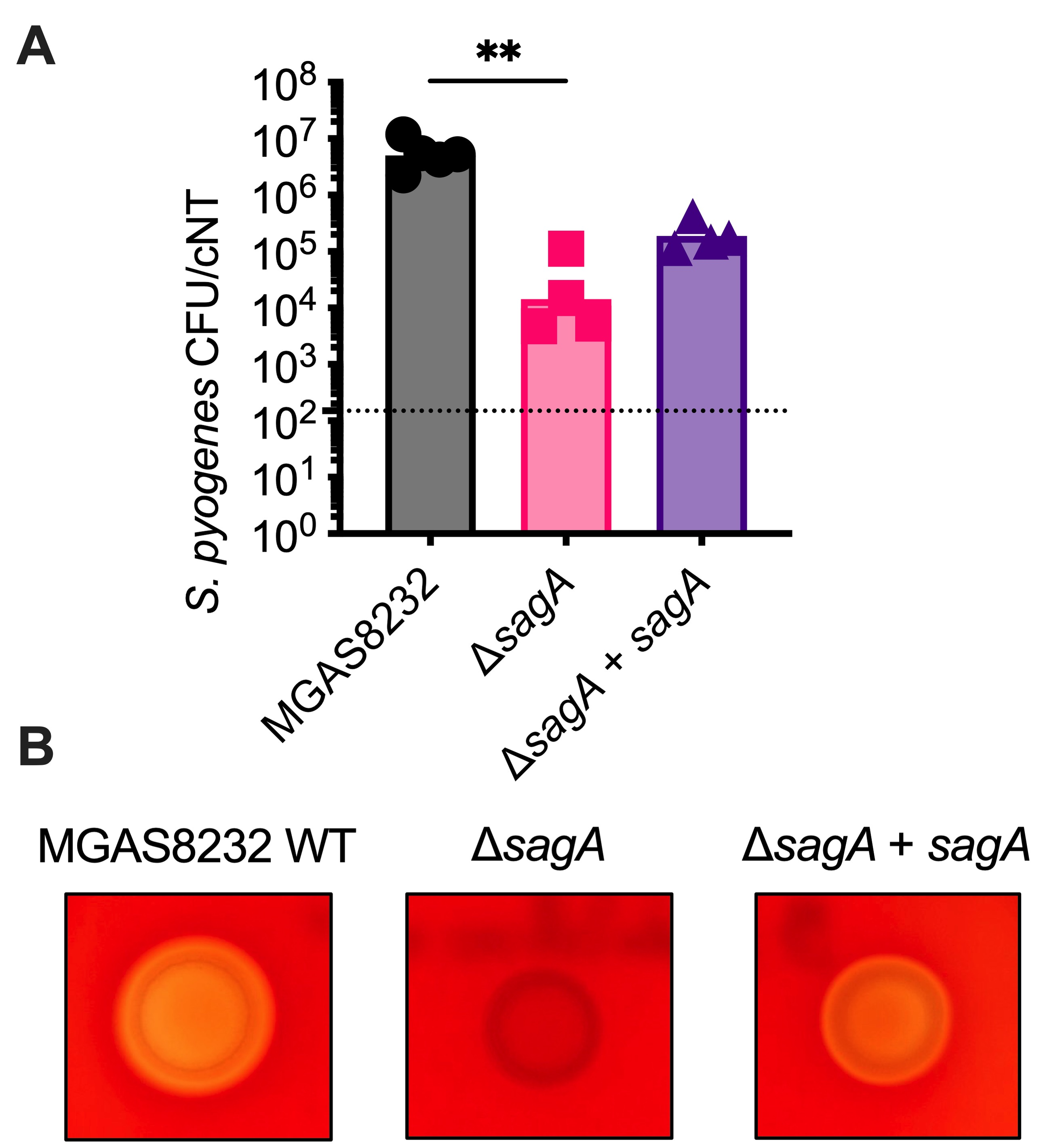

Supplement: S2 Fig — (A) 8–12-week-old B6HLA mice were nasally inoculated with ~1×10^8^ CFU of either the wildtype S. pyogenes MGAS8232, ΔsagA, or ΔsagA + sagA strain. Bacterial burden in the nasal turbinates was determined 48 hours post-inoculation. Data points represent individual mice and bars represent the geometric mean. Dotted line represents the limit of detection (102.17). Statistical analysis was done using a Kruskal-Wallis test and an uncorrected Dunn’s test for multiple comparisons. (B) S. pyogenes MGAS8232 strains grown on TSA with 5% sheep’s blood (** p < 0.01). (TIF) [file ppat.1012072.s002.tif]

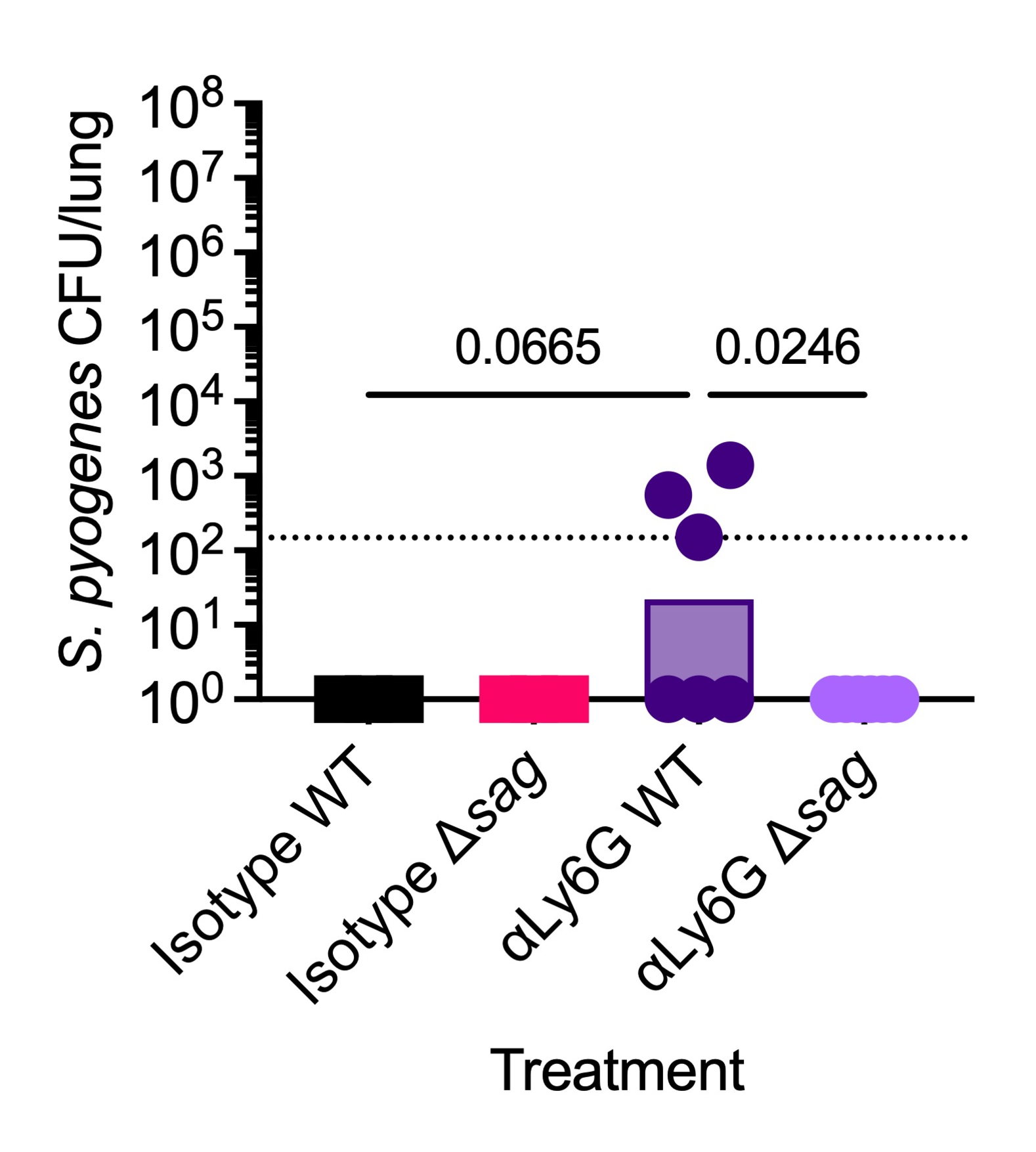

Supplement: S3 Fig — (A) 8–12-week-old B6HLA mice received intraperitoneal injections containing 250 μg of αLy6G antibody or the isotype control 24 hours before infection and 24 hours post-nasopharyngeal infection for a total of 500 μg/mouse. B6HLA mice were infected with S. pyogenes and euthanized 48 hours post-infection. Data points represent individual mice and bars represent the geometric mean. Statistical analysis was done using a Kruskal-Wallis test and an uncorrected Dunn’s test for multiple comparisons. (TIF) [file ppat.1012072.s003.tif]

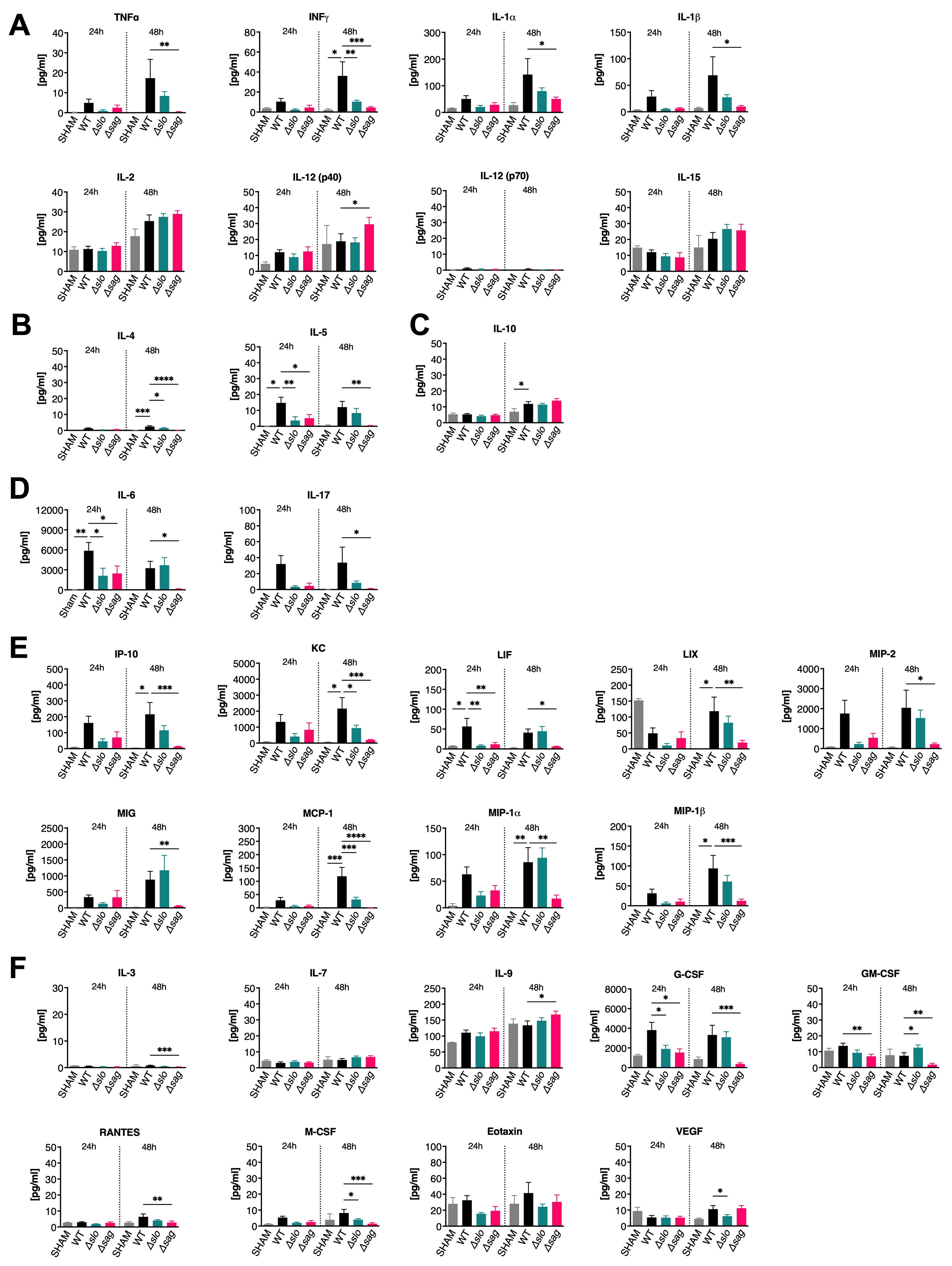

Supplement: S4 Fig — Mice were inoculated with HBSS as sham control or infected intranasally with ~1×10^8^ CFU of S. pyogenes MGAS8232 wildtype, Δslo, or Δsag strains. Mice were euthanized 24 or 48 h post-infection and nasal turbinate homogenates were analyzed using a cytokine array (A) Th1-type cytokines (B) Th2-type cytokines (C) Treg cytokines (D) Th17 cytokines (E) chemokines or (F) growth factors. Data represents the mean ± SEM of nasal turbinate cytokine/chemokine concentrations (n ≥ 3 mice per group). Significance was determined by comparing groups to the wildtype infection at either 24- or 48-hours post-infection using a one-way ANOVA with an uncorrected Fisher’s LSD test (* p < 0.05; ** p < 0.01; *** p < 0.001; **** p < 0.0001). (TIF) [file ppat.1012072.s004.tif]

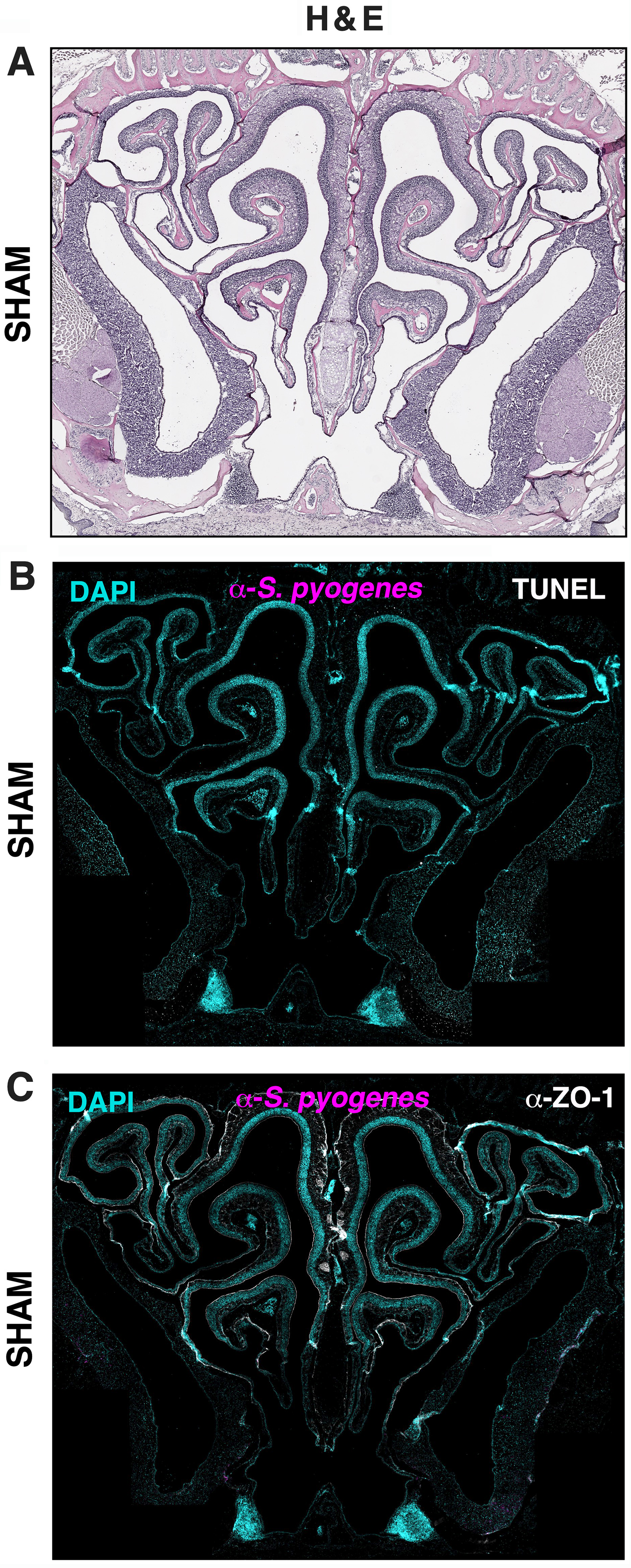

Supplement: S5 Fig — B6HLA mice were intranasally inoculated with HBSS as a sham and euthanized 24 hours post-infection. Heads were fixed in PLP fixation buffer and decalcified with EDTA. Tissues were embedded in OCT, flash frozen, and then sectioned using a cryostat. (A) Nasal turbinates stained with H & E. Images captured at 40X magnification. (B) Nasal turbinates stained with DAPI (teal), fluorescent TUNEL stain for apoptotic and necrotic cells (white) and immunofluorescent S. pyogenes stain (pink). (C) Epithelial barrier staining of nasal turbinates using DAPI (teal), immunofluorescent ZO-1 (white) and S. pyogenes (pink) staining. (B, C) Images captured at 20X magnification. (TIF) [file ppat.1012072.s005.tif]
