# Supplementary material for: Streptolysin S is required for Streptococcus pyogenes nasopharyngeal and skin infection in HLA-transgenic mice
Source: PLoS Pathog. 2024 Mar 7;20(3):e1012072. doi: 10.1371/journal.ppat.1012072 (PMC10950238; doi:10.1371/journal.ppat.1012072)
Supplement: S1 Table — (DOCX) [file ppat.1012072.s006.docx]

| S1 Table. SNPs identified from genome wide comparison to *S. pyogenes* MGAS8232 wildtype strain | | | | | | |
| --- | --- | --- | --- | --- | --- | --- |
| Strain | **Gene** | **Locus Tag** | **Gene length (bp)** | **Nucleotide change** | **Amino acid change** | **Gene product** |
| *S. pyogenes* MGAS8232 Δ*slo* | ktrA | SPYM18_RS01475 | 675 | 643G>A | Glu215Lys | TrkA family potassium uptake protein |
|  | --- | SPYM18_RS09550 | 1290 | 418delA | Ile140fs | Pitrilysin family protein |
| *S. pyogenes* MGAS8232 Δ*sag* | ktrA | SPYM18_RS01475 | 675 | 643G>A | Glu215Lys | TrkA family potassium uptake protein |
|  | lplA | SPYM18_RS05065 | 852 | 265G>A | Val89Ile | Biotin/lipoate A/B protein ligase family protein |
| *S. pyogenes* MGAS8232 Δ*sagA* | ktrA | SPYM18_RS01475 | 675 | 643G>A | Glu215Lys | TrkA family potassium uptake protein |
|  | --- | SPYM18_RS08115 | 1440 | 472C>T | Pro158Ser | Glycoside hydrolase family 32 protein |
|  | --- | SPYM18_RS08775 | 3498 | 3280A>G | Asn1094Asp | Pullulanase |
