# Supplementary material for: Streptolysin S is required for Streptococcus pyogenes nasopharyngeal and skin infection in HLA-transgenic mice
Source: PLoS Pathog. 2024 Mar 7;20(3):e1012072. doi: 10.1371/journal.ppat.1012072 (PMC10950238; doi:10.1371/journal.ppat.1012072)
Supplement: S2 Table — (DOCX) [file ppat.1012072.s007.docx]

| **S2 Table. Primers used in this study** | |
| --- | --- |
| **Primer** | **Primer Sequence (5′ to 3′)^a^** |
| Primers for chromosomal deletions | |
| *slo* upstream for | CCC**GGATCC**CAATATCAAAGAAGGCGATG |
| *slo* upstream rev | CCC**CTGCAG**TTTGTTTCTCATGTAAACCACC |
| *slo* downstream for | CCC**CTGCAG**ACTTATAAGTAGGACTGGTTCAAGAG |
| *slo* downstream rev | CCC**GGTACC**GGCTTTCTTTGCCTTTTTC |
| *sag* upstream for | CCC**GGATCC**AAATGGCTAAAGAAGCTGGATA |
| *sag* upstream rev | CCC**CTGCAG**AAATTTTAACATAAGGTTTACCTC |
| *sag* downstream for | CCC**CTGCAG**ACCAGACTATAATTAGTCTTTTG |
| *sag* downstream rev | CCC**GGTACC**AATATCTCCCTTACTGTATCCT |
| *sagA* upstream for | CCC**GGTACC**GGGAAAATCCCAAGCTTTTT |
| *sagA* upstream rev | CCC**CTGCAG**TTTTAACATAAGGTTTACCTCCTTATC |
| *sagA* downstream for | CCC**CTGCAG**GGTAAATAATCTATTTAGCATCTCTATG |
| *sagA* downstream rev | CCC**GGATCC**CAACGGCAGAATCTGTAAAA |
| Primers for sequencing | |
| M13 for | GTAAAACGACGGCCAGTGAG |
| M13 rev | CAGGAAACAGCTATGACCATG |
| *slo* integration for | AACACCACTTCTTGAAGAAGA |
| *slo* integration rev | GCTGTAACACCTCAAATAGAAT |
| *sag* integration for | TCCTTATCAAAGTTAACCAAATC |
| *sag* integration rev | GTGTGCAGTCACCTGTTGG |
| *sag* integration mid-1 | GATAACATATCCAAGTCTTCCT |
| *sag* integration mid-2 | CTTTGGTTCTGATAATACAGGA |
| *sag* integration mid-3 | TTATTGAAAACGTTTTTTTAAAATAC |
| *sag* integration mid-4 | AATCGTAACACAGAGGCCAAA |
| *sagA* integration for | TGAACATCCAGTCAGCTACG |
| *sagA* integration rev | TGTCGACAGAGCAGTTGATATC |
| ^a^ Restriction endonuclease sites are bolded in the primer sequence. | |
